# Supplementary material for: Phase-separated CCER1 coordinates the histone-to-protamine transition and male fertility
Source: Nat Commun. 2023 Dec 11;14:8209. doi: 10.1038/s41467-023-43480-z (PMC10713660; doi:10.1038/s41467-023-43480-z)
Supplement: Supplementary file 1 — Supplementary information [file 41467_2023_43480_MOESM1_ESM.pdf]

a

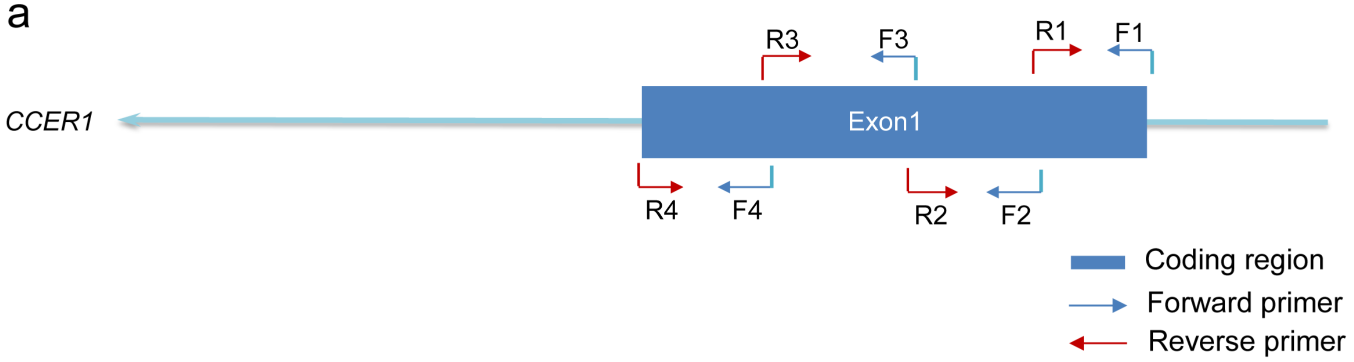

b

Exon		Sequence (5'->3')	Tm (°C)	Product length (bp)
Exon1	F1	GGGAGACCACAGCCAGTATT	60	819
	R1	GCCATACACCCGAAACACTT		
	F2	AAGCAGCCGAAGCAACAG	60	827
	R2	AGCTCCTCCTCTTCCAGCTC		
	F3	GAGGTGTGTGATGCAAAGGA	60	998
	R3	AACCAGTTCCACAGAAGCAAC		
	F4	TTTTCCTCATAGATTTTTCCACCA	60	839
	R4	AATAATGAGAAATGAGAGGCAAGG		

Supplementary Figure 1. Sanger sequencing of the full-length *CCER1* exons. a. Schematic diagram of *CCER1* and the primers designed to sequence the full length of the exon in *CCER1*. The blue rectangle represents the coding region, the blue arrow represents the forward primer and the red arrow represents the reverse primer. b. The sequences (5'->3') of different primers used for Sanger sequencing of the *CCER1* exon, the annealing temperature, and product length for each pair are presented.

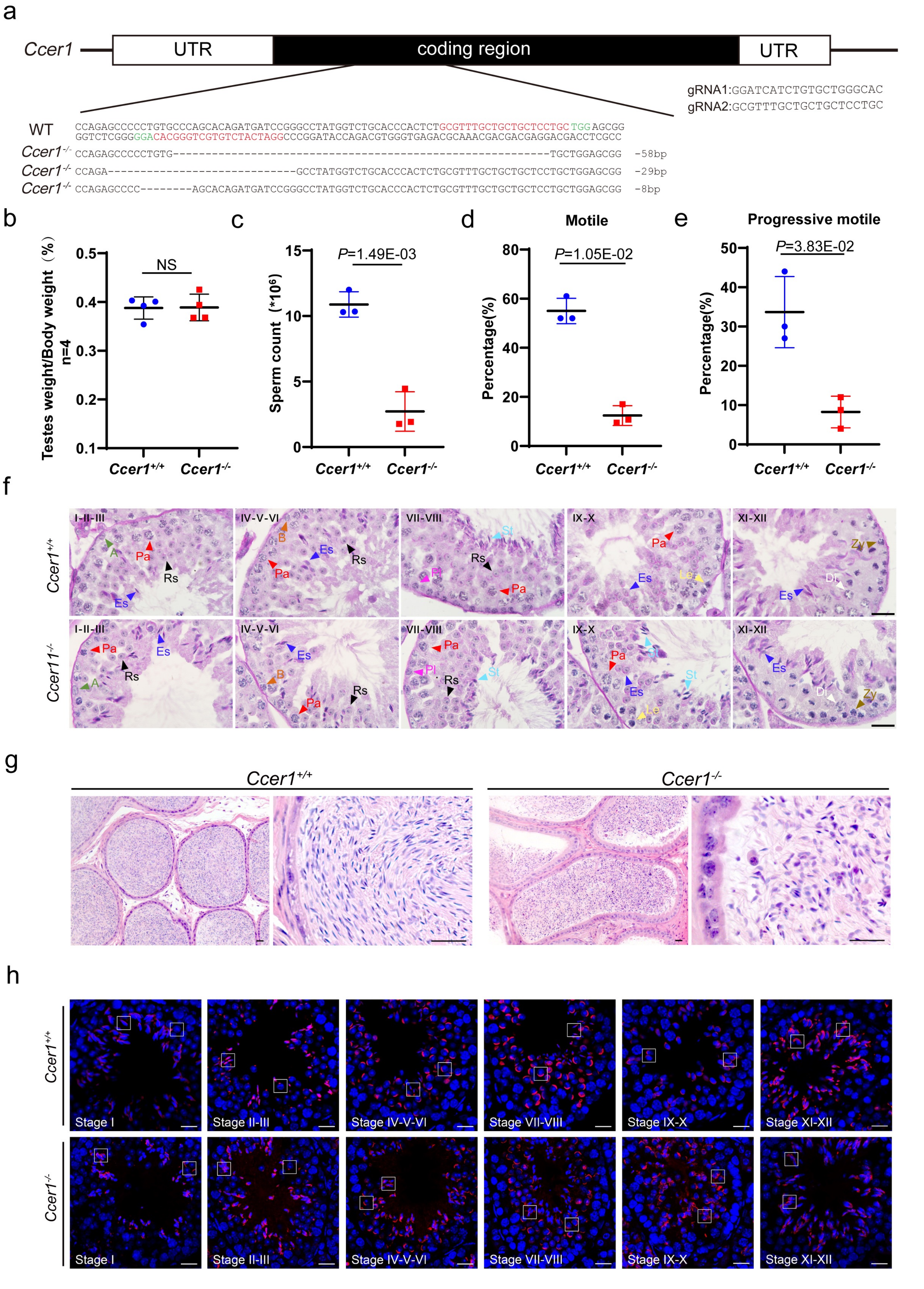
a
Ccer1
UTR
coding region
UTR
gRNA1:GGATCATCTGTGCTGGGCAC
gRNA2:GCGTTTGCTGCTGCTCCTGC
WT CCAGAGCCCCCTGTGCCCAGCACAGATGATCCGGGCCTATGGTCTGCACCCACTCTGCGTTTGCTGCTGCTCCTGCTGGAGCGG
GGTCTCGGGGGACACGGGTCGTGTCTACTAGGCCCGGATACCAGACGTGGGTGAGACGCAAACGACGACGAGGACGACCTCGCC
Ccer1-/- CCAGAGCCCCCTGTG--TGCTGGAGCGG -58bp
Ccer1-/- CCAGA-----------------------------GCCTATGGTCTGCACCCACTCTGCGTTTGCTGCTGCTCCTGCTGGAGCGG -29bp
Ccer1-/- CCAGAGCCCC--------AGCACAGATGATCCGGGCCTATGGTCTGCACCCACTCTGCGTTTGCTGCTGCTCCTGCTGGAGCGG -8bp
b
Testes weight/Body weight (%) n=4
NS
Ccer1+/+
Ccer1-/-
c
Sperm count (*10⁶)
P=1.49E-03
d
Motile
Percentage(%)
P=1.05E-02
e
Progressive motile
Percentage(%)
P=3.83E-02
f
Ccer1+/+
Ccer11-/-
I-II-III
IV-V-VI
VII-VIII
IX-X
XI-XII
A
B
Pa
Pl
Rs
Es
St
Le
Zy
Di
g
Ccer1+/+
Ccer1-/-
h
Ccer1+/+
Ccer1-/-
Stage I
Stage II-III
Stage IV-V-VI
Stage VII-VIII
Stage IX-X
Stage XI-XII

Supplementary Figure 2. Targeting strategy of *Ccer1* deletion in mice and testis pathological analysis. a. Schematic diagram of the targeting strategy at the *Ccer1* locus. sgRNA targeting sequences are red, and PAM cleavage sites are green. b. Statistical comparison of testis/body weight ratio between *Ccer1*$^{+/+}$ and *Ccer1*$^{-/-}$ mice (0.397 % ± 0.022% vs. 0.388 % ± 0.027%, n = 4 for each genotype biological independent mice).Two-sided student's t-test. Error bars, mean ± SD. P = 9.54×10^{-1}. NS denotes no significance. Source data are provided as a Source Data file. c. Statistical comparison of the number spermatozoa in *Ccer1*$^{+/+}$ and *Ccer1*$^{-/-}$ mouse epididymis (10.88 ± 0.97×10^{6}/ml vs. 2.71 ± 1.51×10^{6}/ml, n = 3 for each genotype biological independent mice). Two-sided student's t-test. Error bars, mean ± SD. P = 1.49×10^{-3}. Source data are provided as a Source Data file. d. Percentage of motile sperm in *Ccer1*$^{+/+}$ and *Ccer1*$^{-/-}$ mouse epididymis (55.0% ± 5.20% vs. 12.4% ± 4.01%, n = 3 for each genotype biological independent mice). Two-sided student's t-test. Error bars, mean ± SD. P = 1.05×10^{-2}. Source data are provided as a Source Data file. e. Percentage of progressively motile sperm in *Ccer1*$^{+/+}$ and *Ccer1*$^{-/-}$ mouse epididymis (33.7% ± 9.07% vs. 8.25 % ± 4.02%, n = 3 for each genotype biological independent mice). Two-sided student's t-test. Error bars, mean ± SD. P = 3.83×10^{-2}. Source data are provided as a Source Data file. f. Periodic acid-Schiff (PAS) staining of sections in adult *Ccer1* $^{+/+}$ and *Ccer1* $^{-/-}$ mouse testes. Scale bar: 20 μm. g. Representative images of Hematoxylin and Eosin (H&E)-stained cross- sections of epididymis from adult *Ccer1*$^{+/+}$ and *Ccer1*$^{-/-}$ mice. Scale bar: 20 μm. h. Representative images of PNA staining of sections in adult *Ccer1* $^{+/+}$ and *Ccer1* $^{-/-}$ mouse testes. Scale bar: 20 μm. Highlighted spermatids in white frames were shown in Figure 3d.

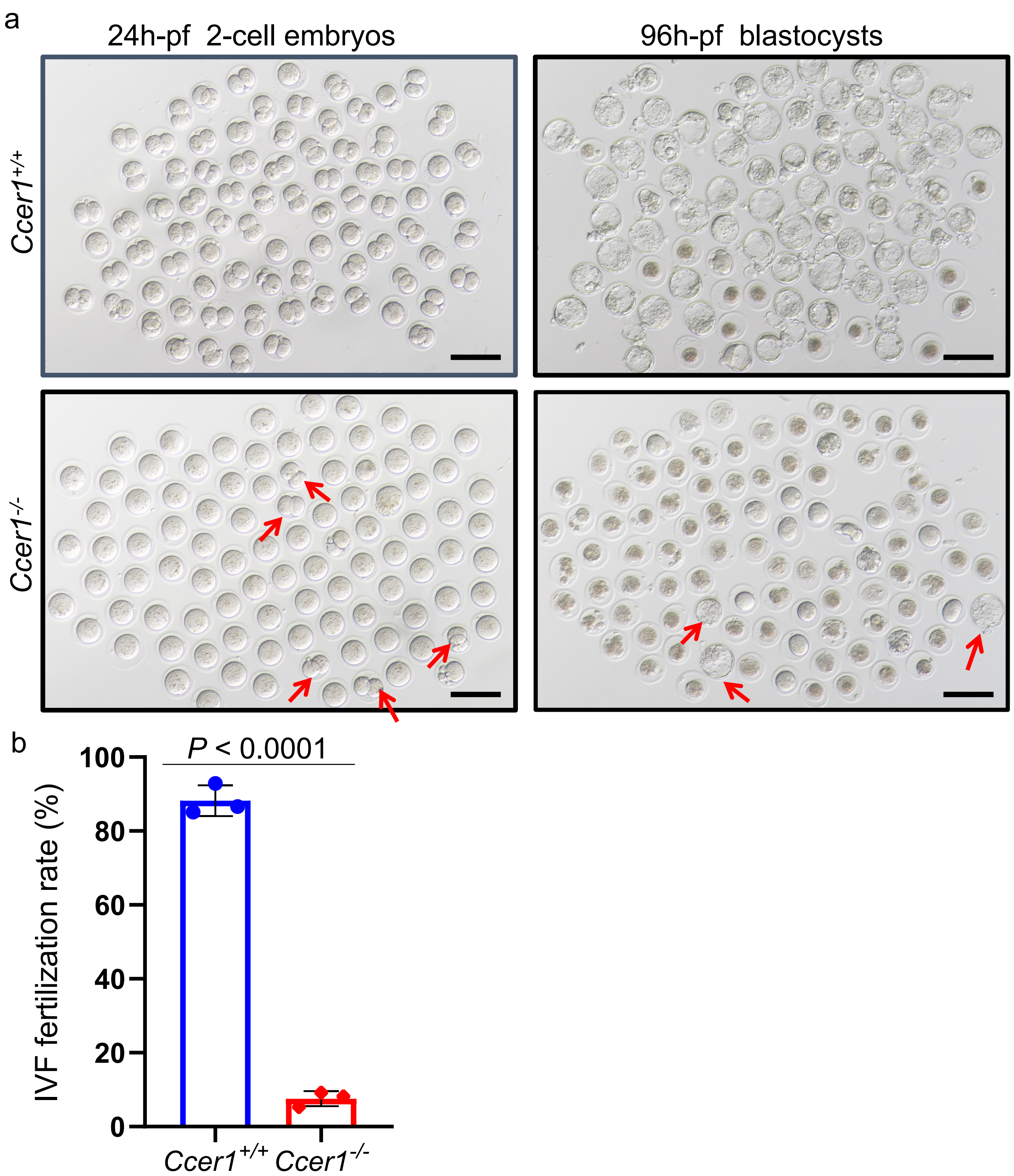

Supplementary Figure 3. *In vitro* fertilization (IVF) assay. a. Representative DIC imaging of embryos cultured in vitro. Sperm were collected from *Ccer1*$^{+/+}$ and *Ccer1*$^{-/-}$ mice respectively. Scale bar: 20 μm. b. Statistical comparison of fertilization rate between *Ccer1*$^{+/+}$ and *Ccer1*$^{-/-}$ mice (88.2 % ± 4.1% vs. 7.5 % ± 2.0%, Littermates of each genotype underwent three biologically independent IVF experiments). Two-sided student's t-test. Error bars, mean ± SD. P < 0.0001.Red arrows indicate fertilized eggs. Source data are provided as a Source Data file. Abbreviations: h-pf (hours post fertilization)

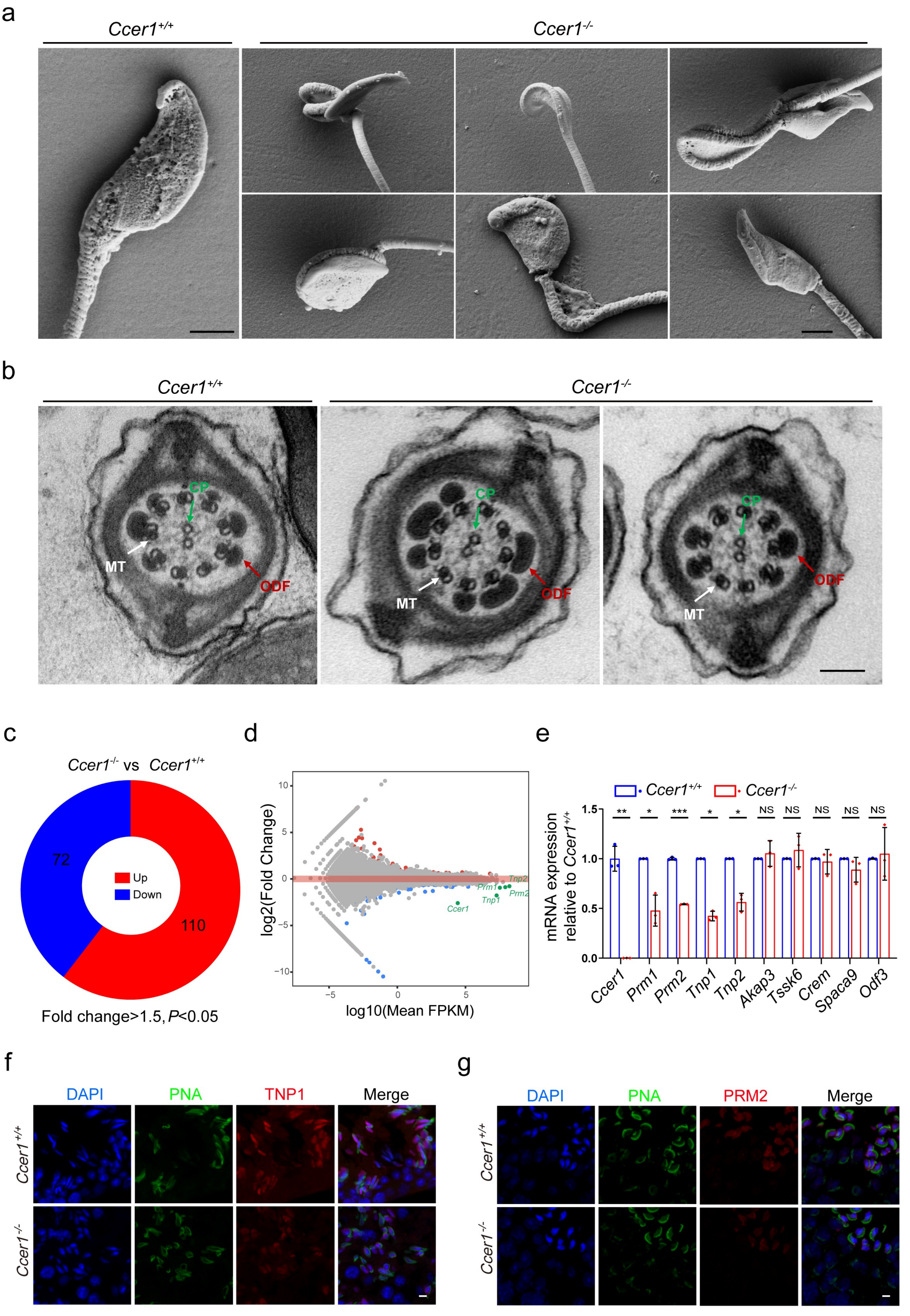
a
Ccer1+/+
Ccer1-/-
b
Ccer1+/+
Ccer1-/-
CP
MT
ODF
c
Ccer1-/- vs Ccer1+/+
72
110
Up
Down
Fold change>1.5, P<0.05
d
log2(Fold Change)
log10(Mean FPKM)
Tnp2
Prm1
Prm2
Tnp1
Ccer1
e
Ccer1+/+
Ccer1-/-
mRNA expression relative to Ccer1+/+
** * *** * * NS NS NS NS NS
Ccer1
Prm1
Prm2
Tnp1
Tnp2
Akap3
Tssk6
Crem
Spaca9
Odf3
f
DAPI
PNA
TNP1
Merge
Ccer1+/+
Ccer1-/-
g
DAPI
PNA
PRM2
Merge
Ccer1+/+
Ccer1-/-

Supplementary Figure 4. RNA seq and immunofluorescence analysis on *Ccer1* $^{-/-}$ mouse testis. a. Scanning electron microscopy (SEM) analysis of mature sperm in adult *Ccer1* $^{+/+}$ and *Ccer1* $^{-/-}$ mice. The sperm heads of *Ccer1* $^{-/-}$ mice were abnormal. Scale bar: 5 μm. b. Transmission electron microscopy (TEM) shows the ultrastructure of mature sperms from adult *Ccer1*$^{+/+}$ and *Ccer1*$^{-/-}$ mice. The typical "9+2" microtubule structure was integrated. Scale bar: 200 nm. c. Number of significant (fold change >1.5, P < 0.05) up- or downregulation genes in *Ccer1*$^{-/-}$ vs *Ccer1*$^{+/+}$ adult testis. n=3 for each genotype. d. Volcano Plot showing differentially expressed genes between *Ccer1*$^{+/+}$ and *Ccer1*$^{-/-}$ mouse testes as identified by RNA deep-sequencing. e. Quantitative reverse transcription PCR (qRT-PCR) validating the spermatogenesis-associated mRNA levels between *Ccer1*$^{+/+}$ and *Ccer1*$^{-/-}$ mouse testes. Expression levels were normalized geometric mean of beta (β)-actin. Error bars correspond to means ± SD (n=3 for each genotype biological independent mice, NS denotes no significance, *denotes P < 0.05, **denotes P < 0.01, ***denotes P < 0.001). Two-sided student's t-test. Error bars, mean ± SD. $P = 5.12\times10^{-3}$ (for *Ccer1*), $P = 2.87\times10^{-2}$ (for *Prm1*), $P = 4.54\times10^{-4}$ (for *Prm2*), $P = 2.34\times10^{-3}$ (for *Tnp1*), $P = 1.36\times10^{-2}$ (for *Tnp2*), $P = 5.49\times10^{-1}$ (for *Akap3*), $P = 4.70\times10^{-1}$ (for *Tssk6*), $P = 7.17\times10^{-1}$ (for *Crem*), $P = 2.64\times10^{-1}$ (for *Spaca9*), $P = 7.77\times10^{-1}$ (for *Odf3*). Source data are provided as a Source Data file. f. Immunofluorescence comparison of TNP1 between *Ccer1*$^{+/+}$ and *Ccer1*$^{-/-}$ mouse testes. Scale bar: 5 μm. g. Immunofluorescence comparison of Protamine 2 between *Ccer1*$^{+/+}$ and *Ccer1*$^{-/-}$ mouse testes. Scale bar: 5 μm.

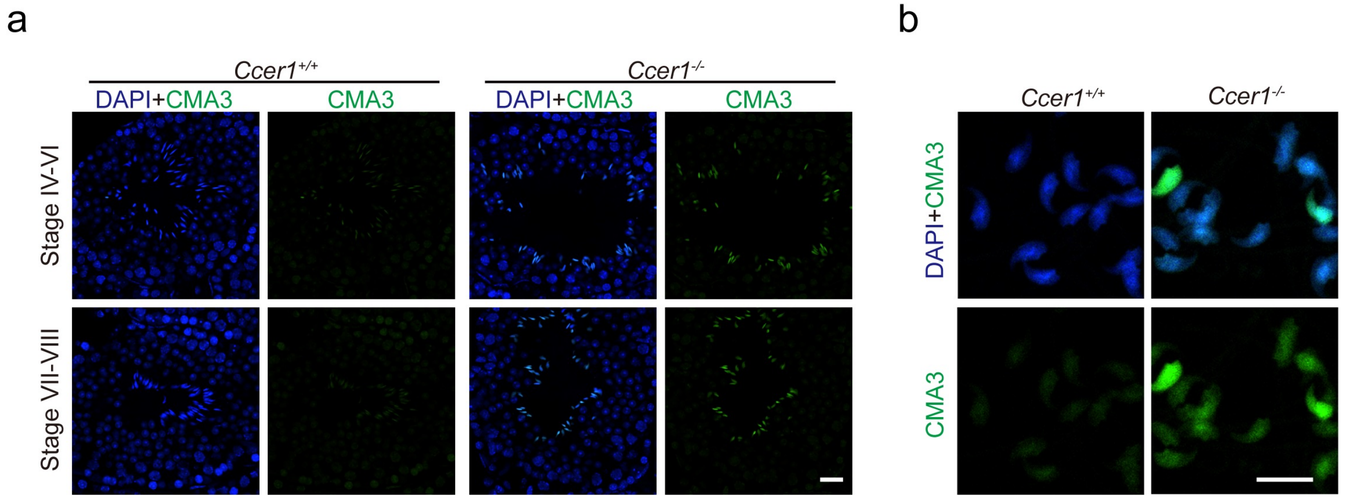

c

Rep1	Fq	mapped	unique mapped	valid_interaction	valid_interaction_rmdup	trans_interaction	cis_interaction	cis_longRange	Intra-fragment	Mapping rate	Fraction of "Hi-C contacts"	Intra-fragment rate	Intra Long Range rate*	Ligation
mouse_sperm_WT_rep1	43021327	36624485	23300823	20778972	13725837	7133734	6592103	6169989	1916184	85.13%	48.30%	8.22%	26.48%	48.28%
mouse_sperm_WT_rep2	74775454	64793977	42990969	38282466	20300582	11244351	9056231	7907617	3699627	86.65%	51.20%	8.61%	18.39%	41.13%
mouse_sperm_KO_rep1	50921001	44096059	28908180	23142996	17034195	7465898	9568297	8690090	4675579	86.60%	45.45%	16.20%	30.06%	42.89%
mouse_sperm_KO _rep2	82511134	61121772	36961598	25093003	19286806	9649234	9637572	8719154	9821024	74.08%	30.41%	26.50%	23.59%	24.92%
mouse_sperm_KO_rep3	62663398	50463553	32724045	22010807	15874578	7943685	7930893	6831107	8379568	80.53%	35.13%	25.60%	20.87%	23.53%
Ke et al.	47806541	45433716	26804287	24840332	24559603	7431346	17128257	15191487	1817524	95.04%	51.96%	6.78%	56.68%	49.39%
Jung et al.	75000000	73885310	44906774	23020264	20667912	7182165	13485747	11480786	10158737	98.51%	30.69%	22.62%	25.57%	47.41%

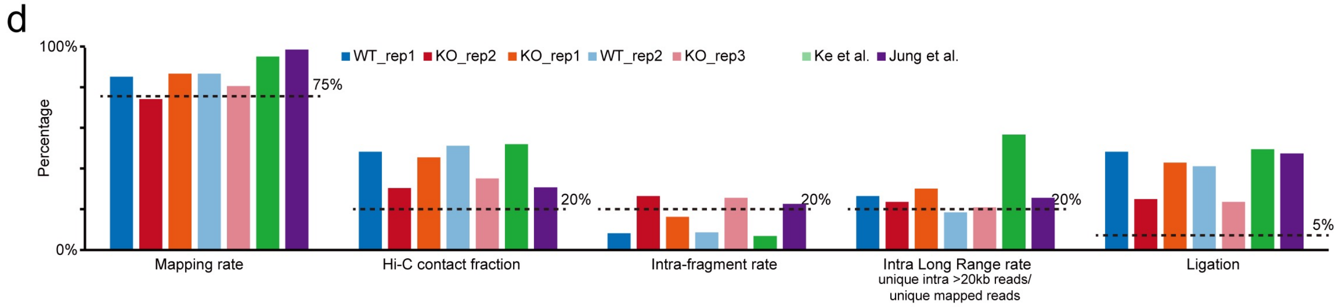

Supplementary Figure 5. CMA3 immunofluorescence staining and Hi-C sequencing information. a. CMA3 immunofluorescence staining in *Ccer1*$^{+/+}$ and *Ccer1*$^{-/-}$ mouse testis. Scale bar: 20 μm. b. CMA3 immunofluorescence staining in *Ccer1*$^{+/+}$ and *Ccer1*$^{-/-}$ mouse sperm. Scale bar: 10 μm. c. Sequencing information of mouse sperm Hi-C experiments among our study and others [1,2]. d. Bar graphs showing the Hi-C quality control parameters in mouse sperm Hi-C datasets.

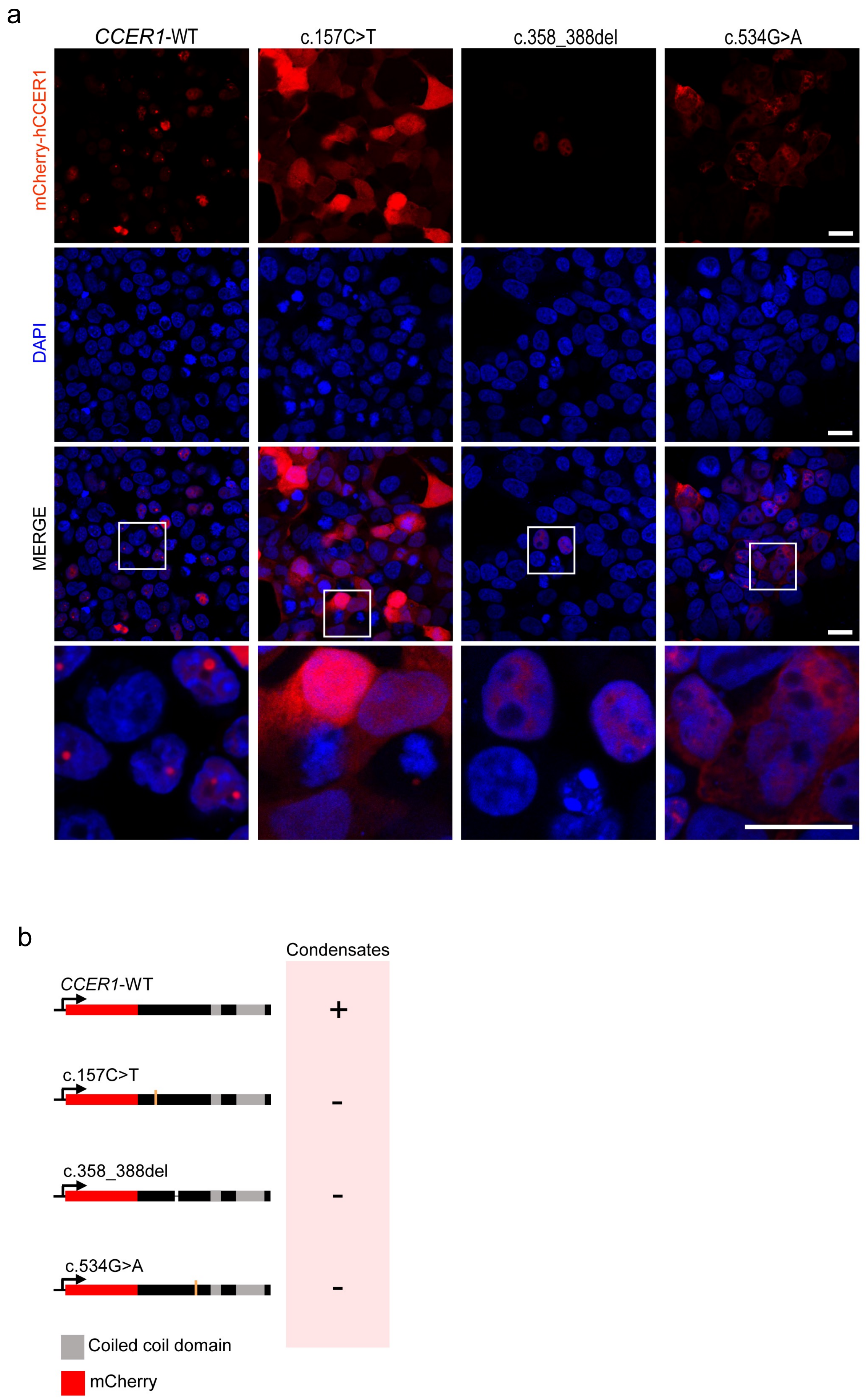
a
CCER1-WT
c.157C>T
c.358_388del
c.534G>A
mCherry-hCCER1
DAPI
MERGE
b
Condensates
CCER1-WT
+
c.157C>T
-
c.358_388del
-
c.534G>A
-
Coiled coil domain
mCherry

Supplementary Figure 6. Mutations led to CCER1 LLPS deficiency. a. Ectopically overexpressed mCherry fusion WT and mutant hCCER1 proteins in HEK 293T cells. Scale bar: 5μm. b. Mutations in detail. All mutations led to CCER1 LLPS deficiency.

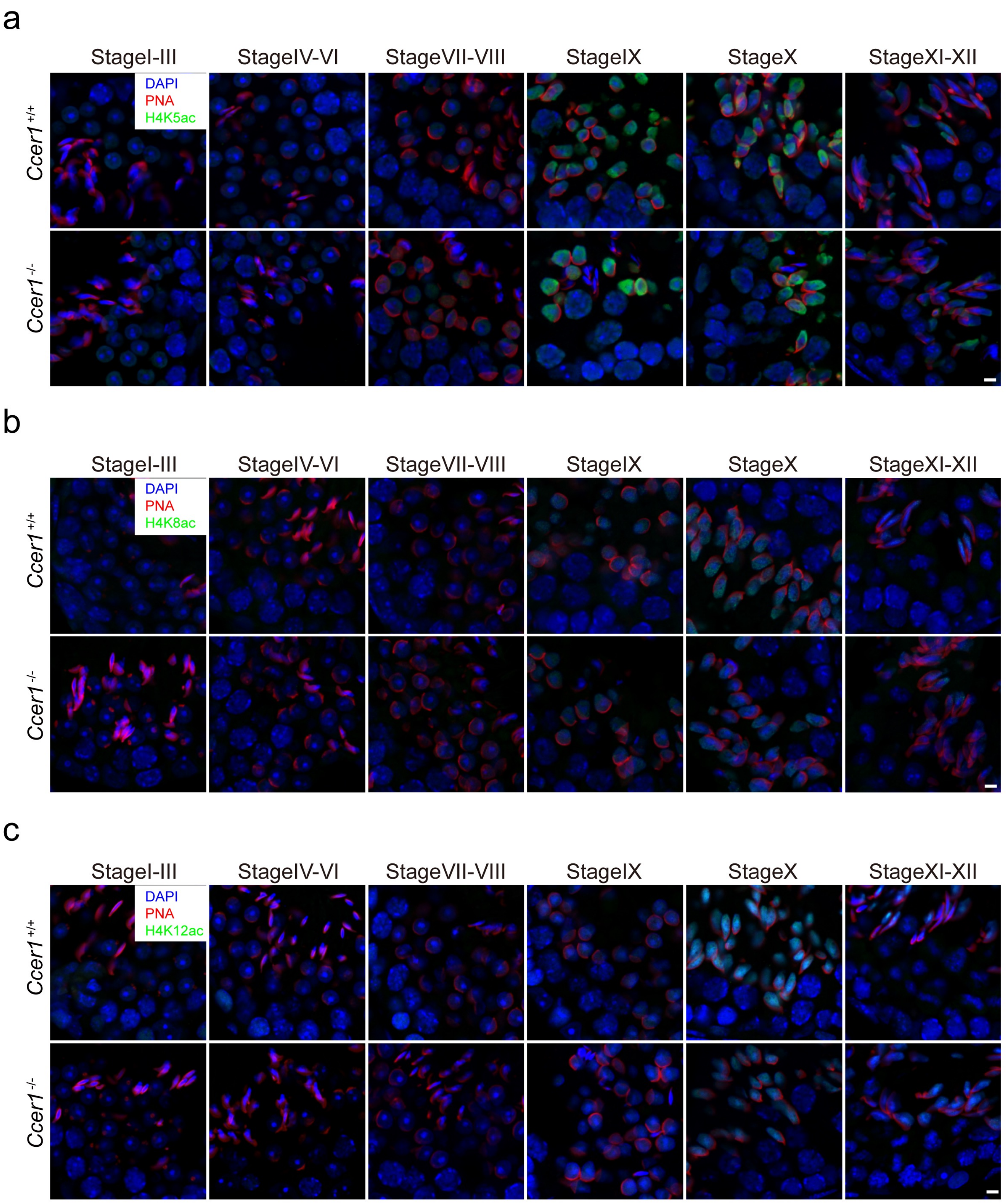
a
StageI-III
StageIV-VI
StageVII-VIII
StageIX
StageX
StageXI-XII
DAPI
PNA
H4K5ac
Ccer1 +/+
Ccer1 -/-
b
StageI-III
StageIV-VI
StageVII-VIII
StageIX
StageX
StageXI-XII
DAPI
PNA
H4K8ac
Ccer1 +/+
Ccer1 -/-
c
StageI-III
StageIV-VI
StageVII-VIII
StageIX
StageX
StageXI-XII
DAPI
PNA
H4K12ac
Ccer1 +/+
Ccer1 -/-

Supplementary Figure 7. Immunofluorescence of the acetylation of histone H4 in mouse testes. a. Immunofluorescence comparison of H4K5ac between *Ccer1*$^{+/+}$ and *Ccer1*$^{-/-}$ mouse testes. Scale bar: 5 μm. b. Immunofluorescence comparison of H4K8ac between*Ccer1*$^{+/+}$ and *Ccer1*$^{-/-}$ mouse testes. Scale bar: 5 μm. c. Immunofluorescence comparison of H4K12ac between *Ccer1*$^{+/+}$ and *Ccer1*$^{-/-}$ mouse testes. Scale bar: 5 μm.

Figure 1d

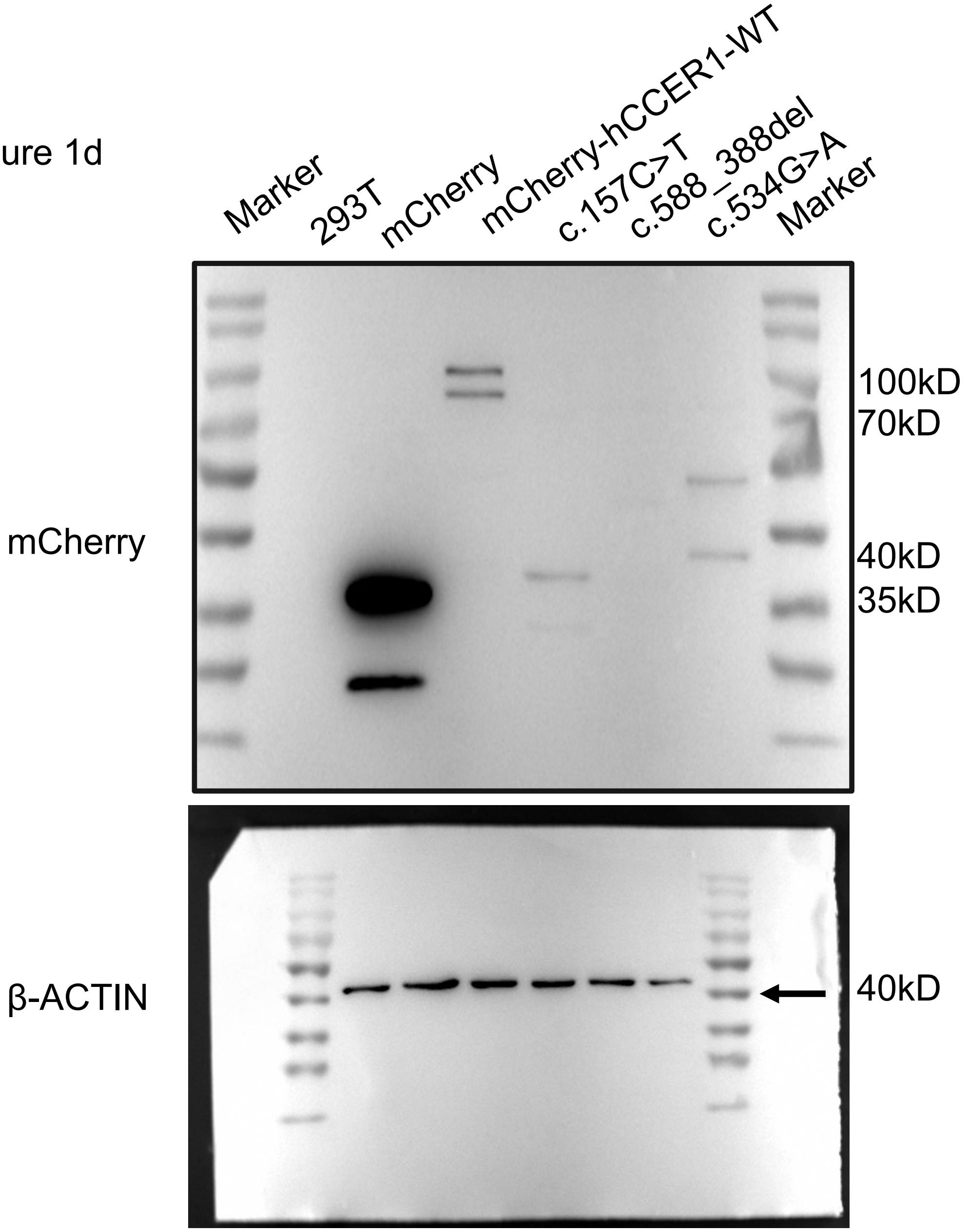

Figure 2a

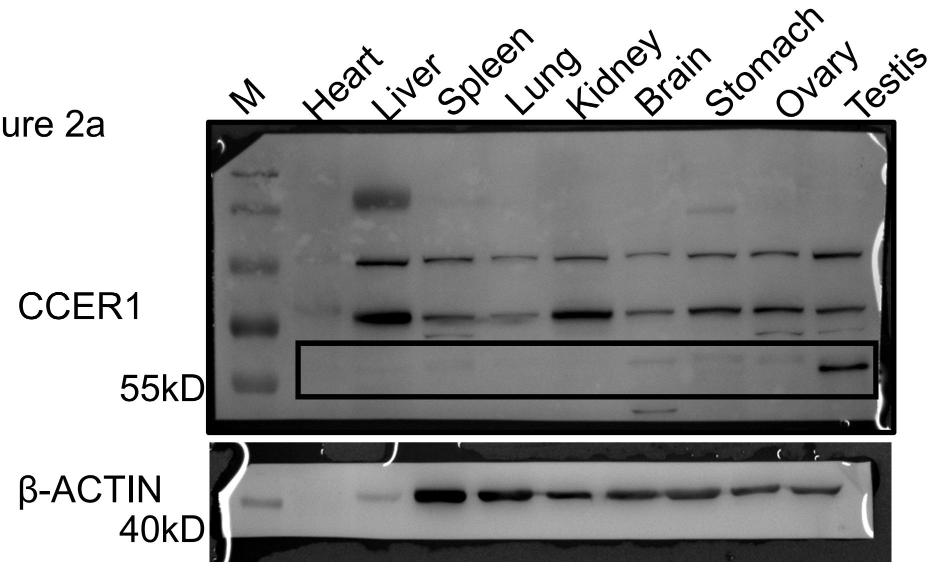

Supplementary Figure 8. Unprocessed images of immunoblotting

PVDF membranes were cut into several small pieces to incubate with different antibodies for immunoblotting. Black boxes indicate images showed in relevant figures.

Figure 2b

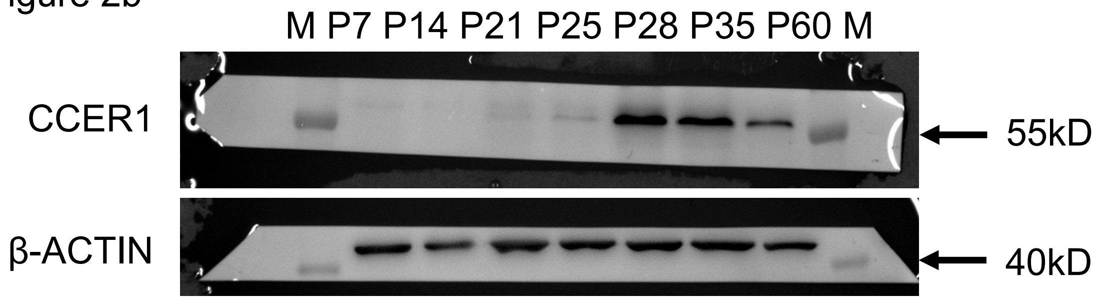

Figure 3b

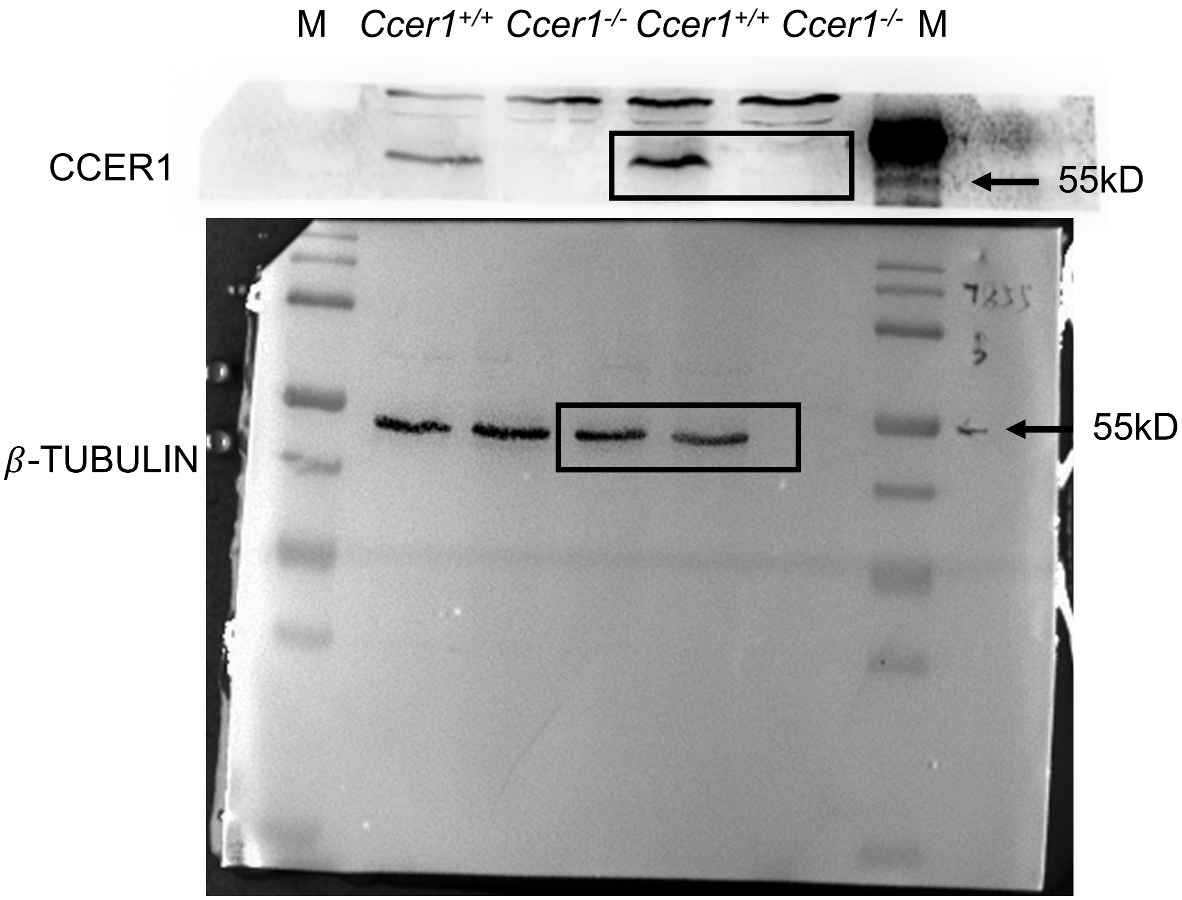

Supplementary Figure 8. Unprocessed images of immunoblotting
PVDF membranes were cut into several small pieces to incubate with different antibodies for immunoblotting. Black boxes indicate images showed in relevant figures.

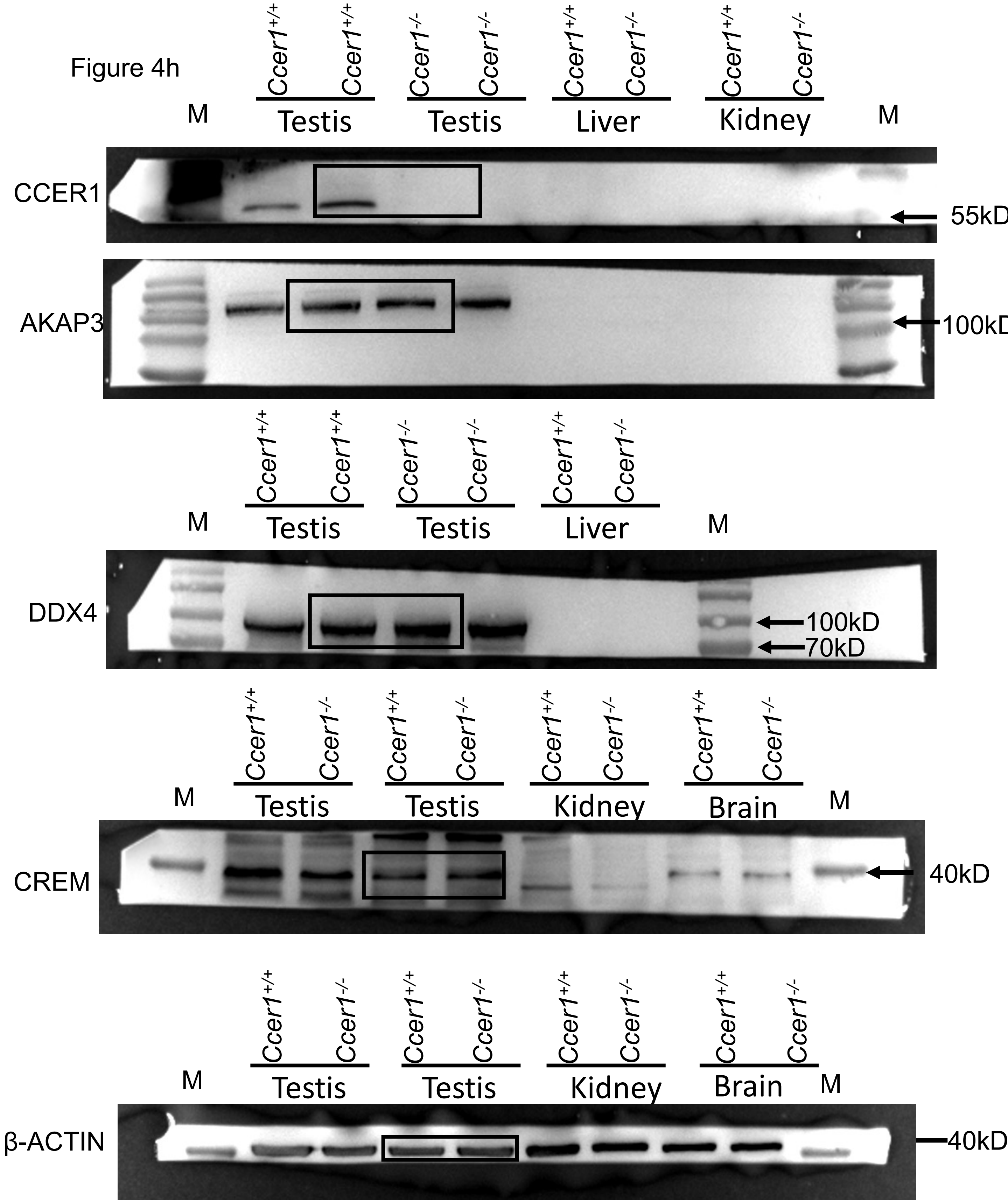

Supplementary Figure 8. Unprocessed images of immunoblotting
PVDF membranes were cut into several small pieces to incubate with different antibodies for immunoblotting. Black boxes indicate images showed in relevant figures.

Figure 4i

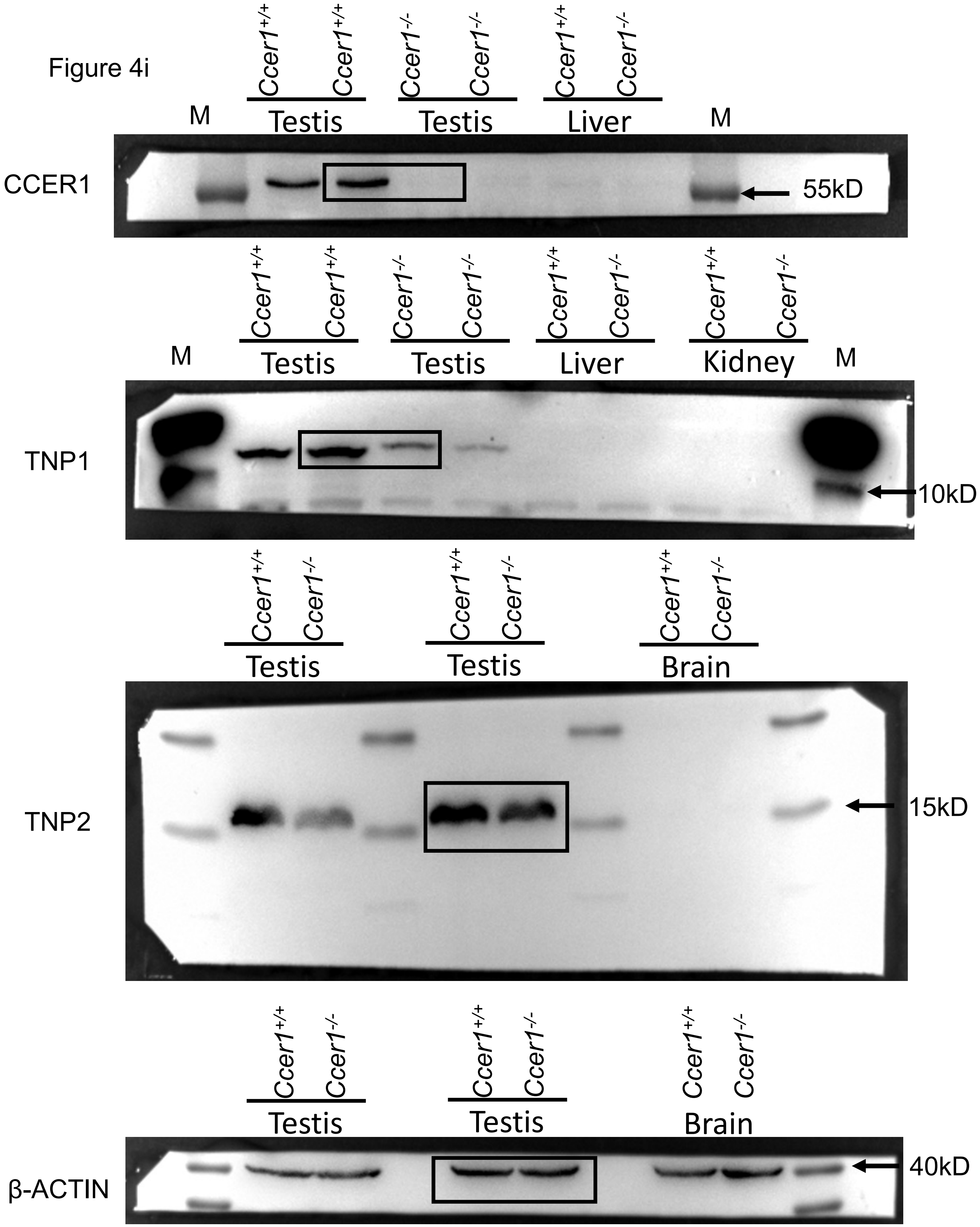

Supplementary Figure 8. Unprocessed images of immunoblotting

PVDF membranes were cut into several small pieces to incubate with different antibodies for immunoblotting. Black boxes indicate images showed in relevant figures.

Figure 4j

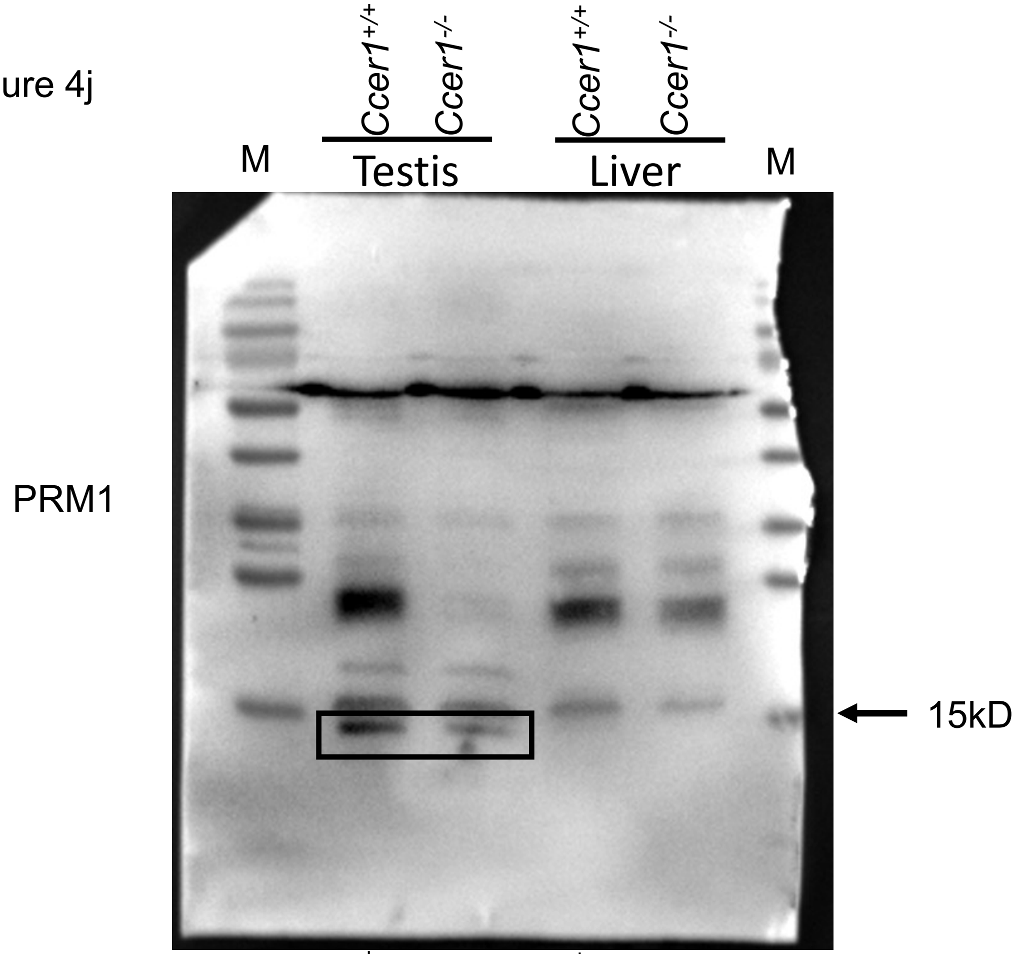

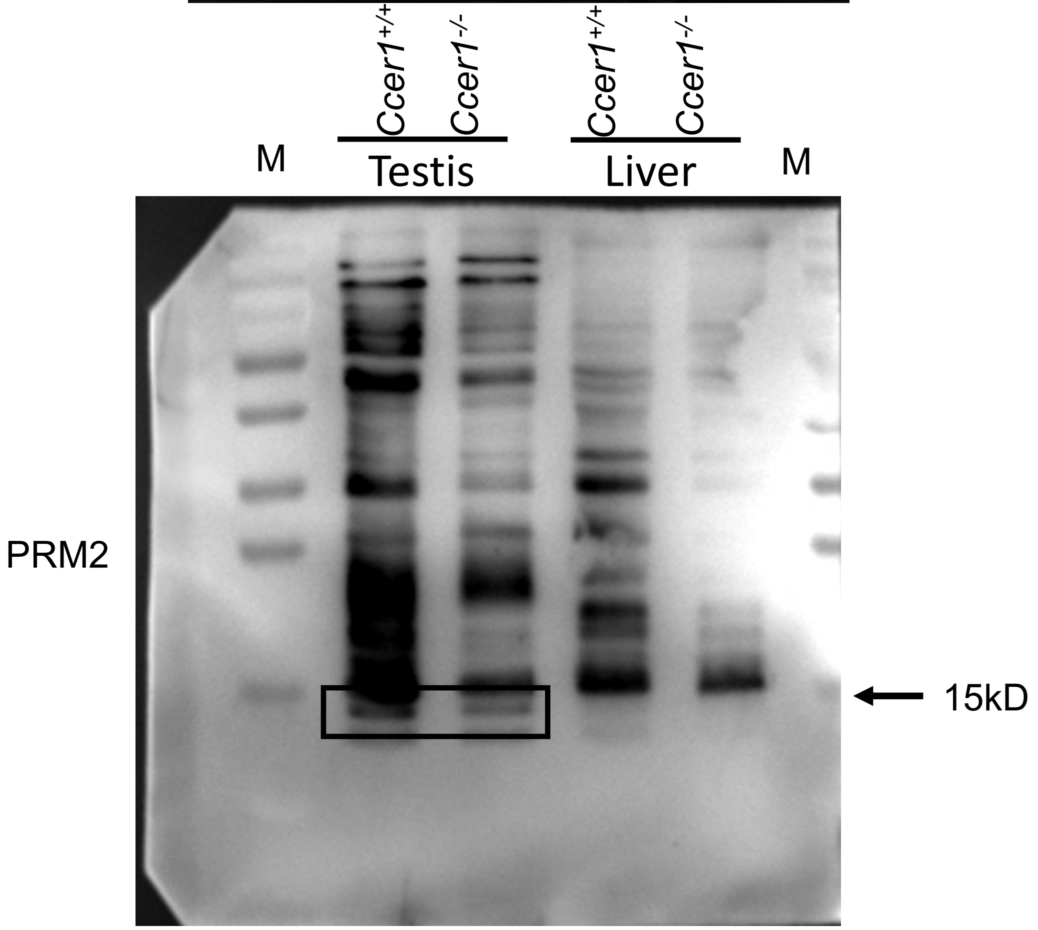

Supplementary Figure 8. Unprocessed images of immunoblotting

PVDF membranes were cut into several small pieces to incubate with different antibodies for immunoblotting. Black boxes indicate images showed in relevant figures.

Figure 4j

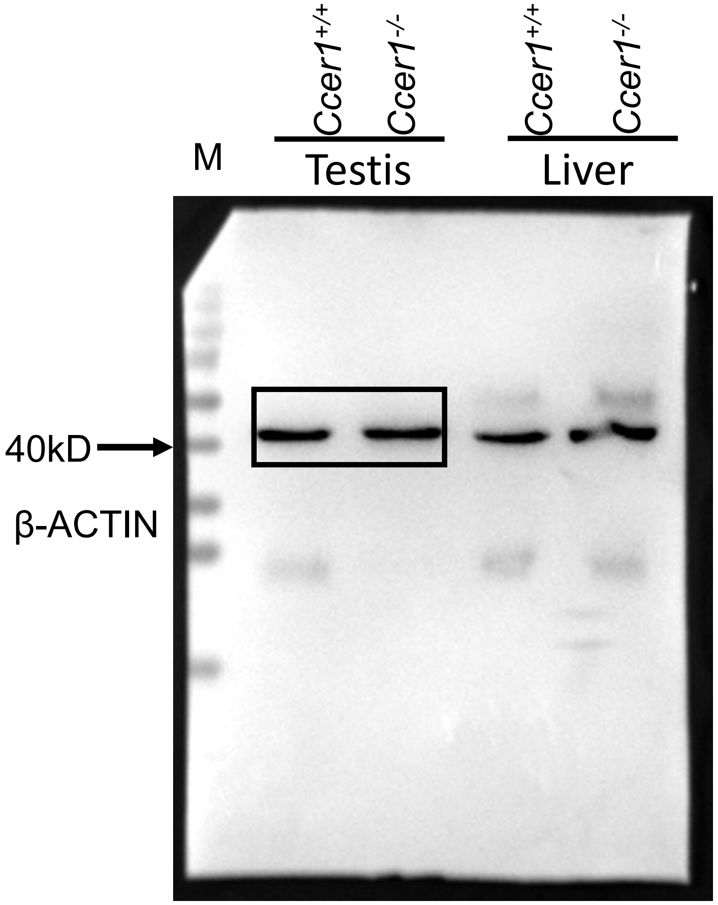

Figure 4k

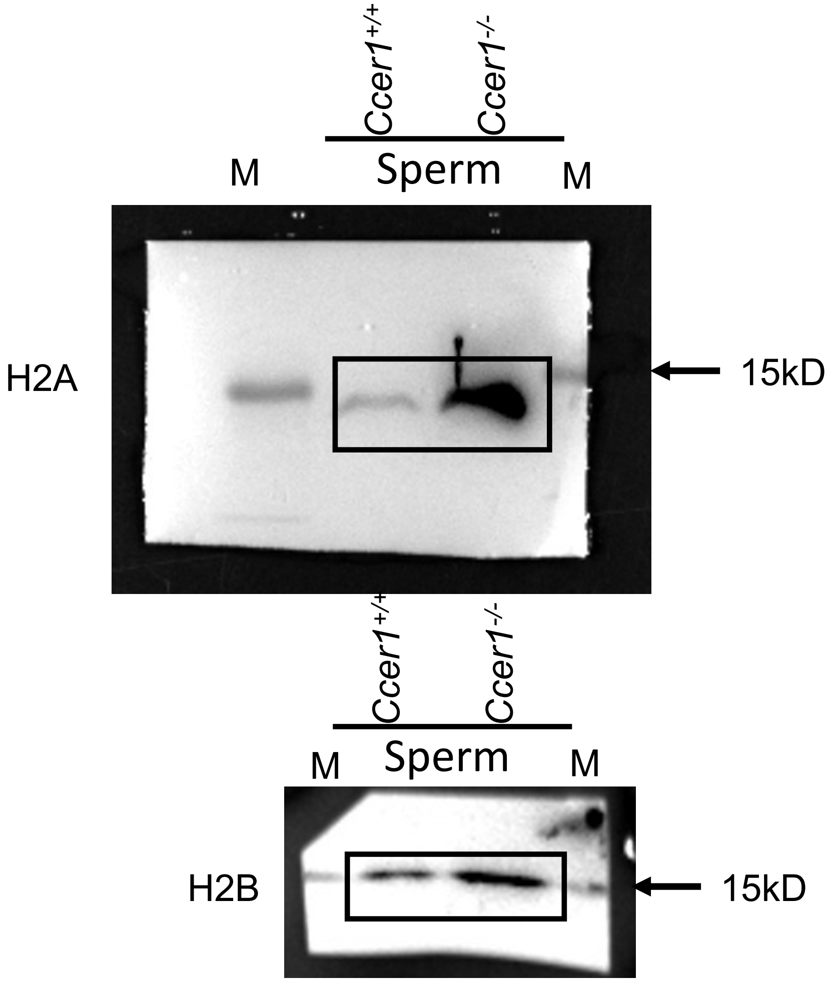

Supplementary Figure 8. Unprocessed images of immunoblotting

PVDF membranes were cut into several small pieces to incubate with different antibodies for immunoblotting. Black boxes indicate images showed in relevant figures.

Figure 4k

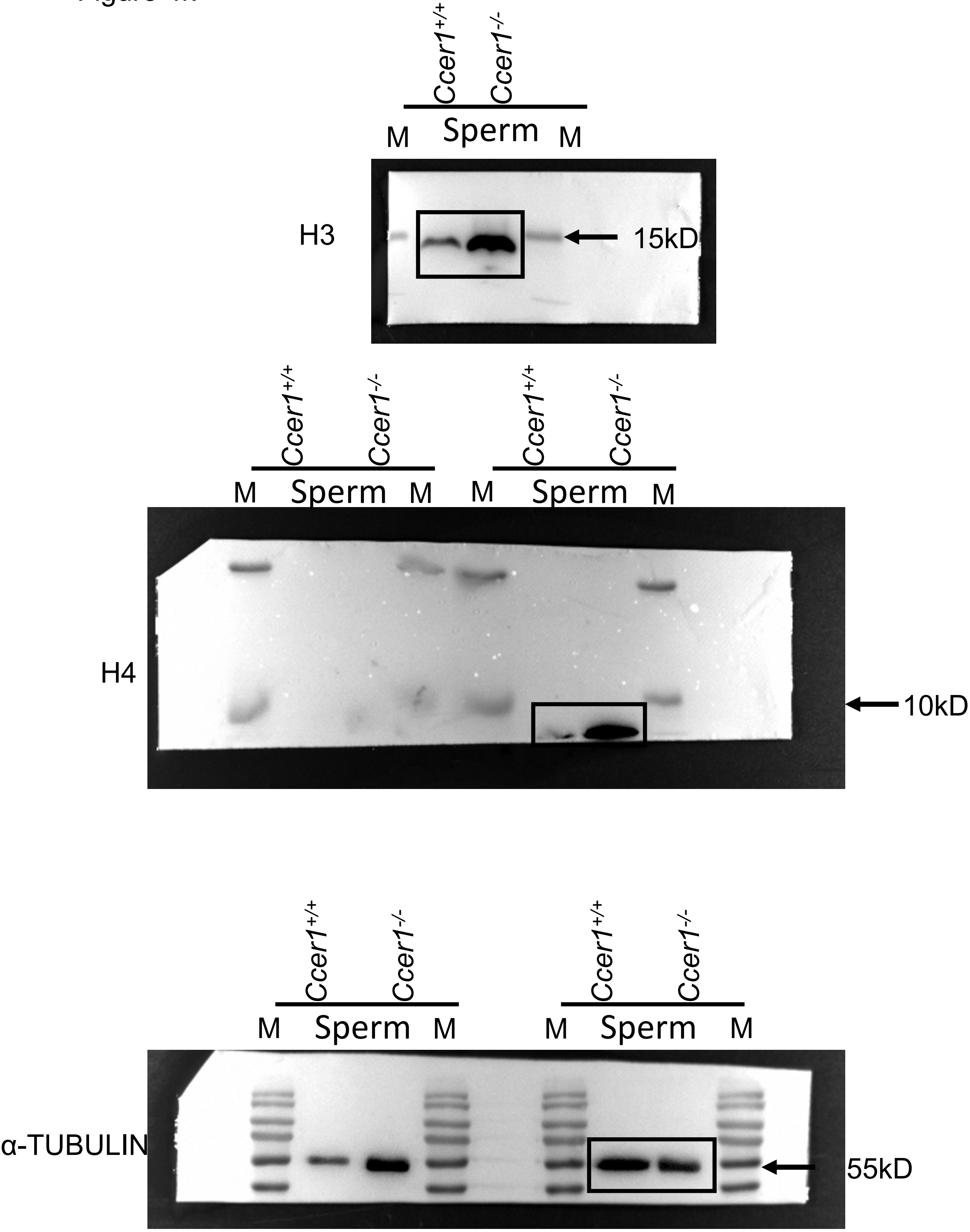

Supplementary Figure 8. Unprocessed images of immunoblotting
PVDF membranes were cut into several small pieces to incubate with different antibodies for immunoblotting. Black boxes indicate images showed in relevant figures.

Figure 4l

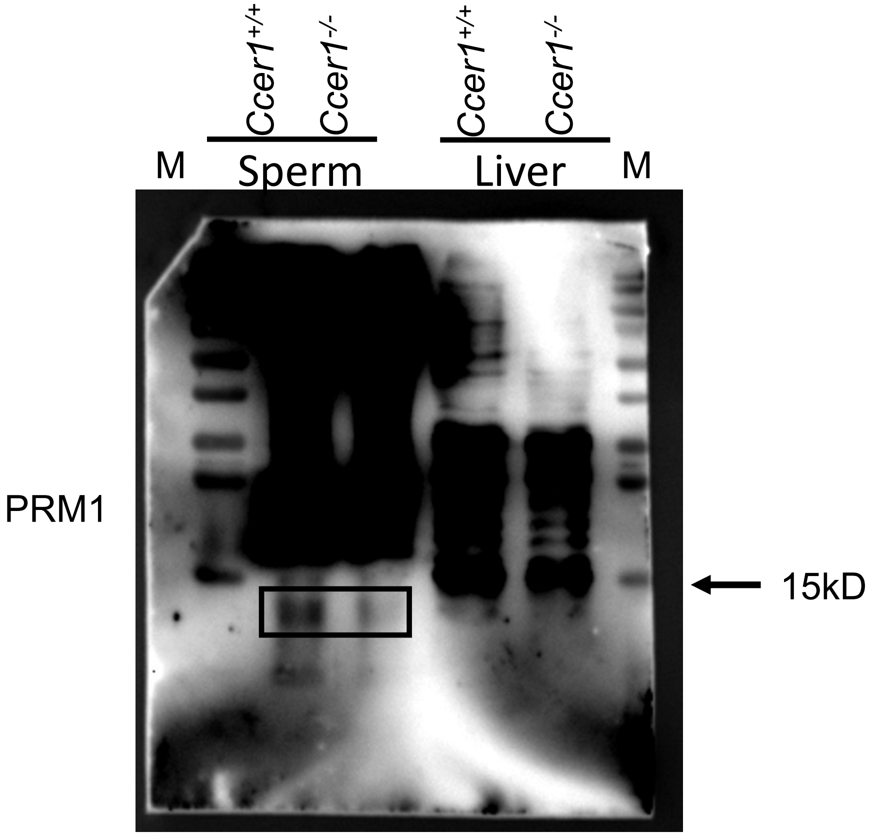

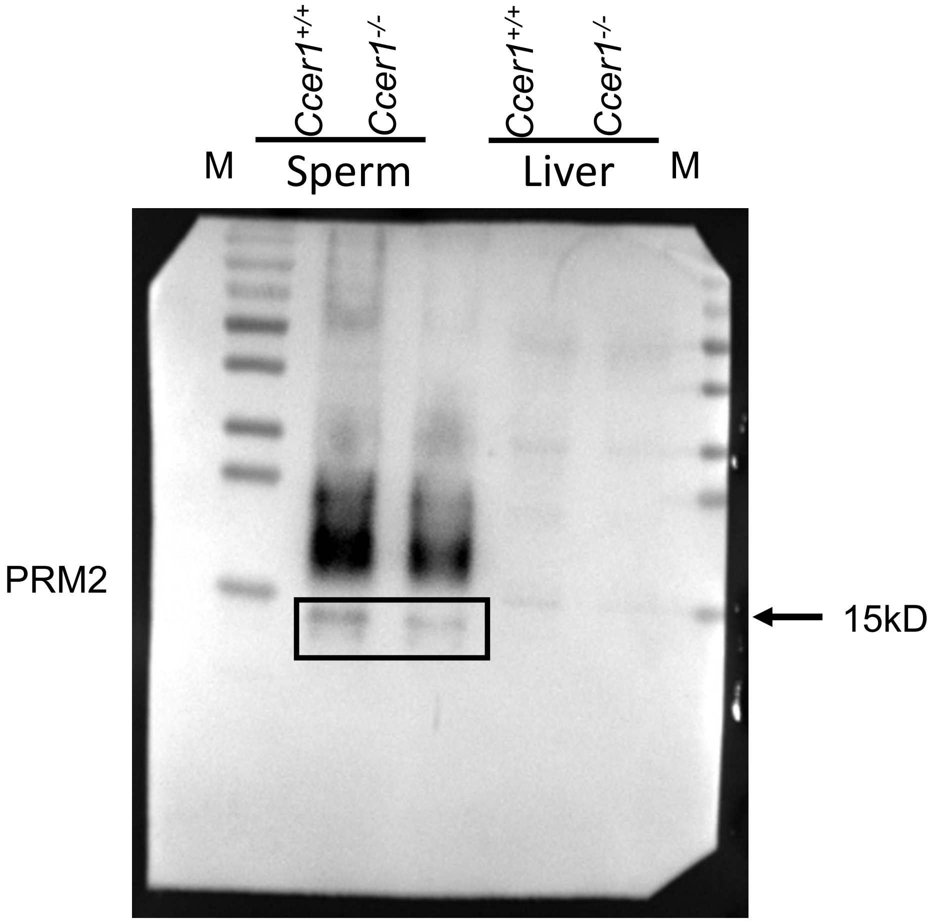

Supplementary Figure 8. Unprocessed images of immunoblotting
PVDF membranes were cut into several small pieces to incubate with different antibodies for immunoblotting. Black boxes indicate images showed in relevant figures.

Figure 5c

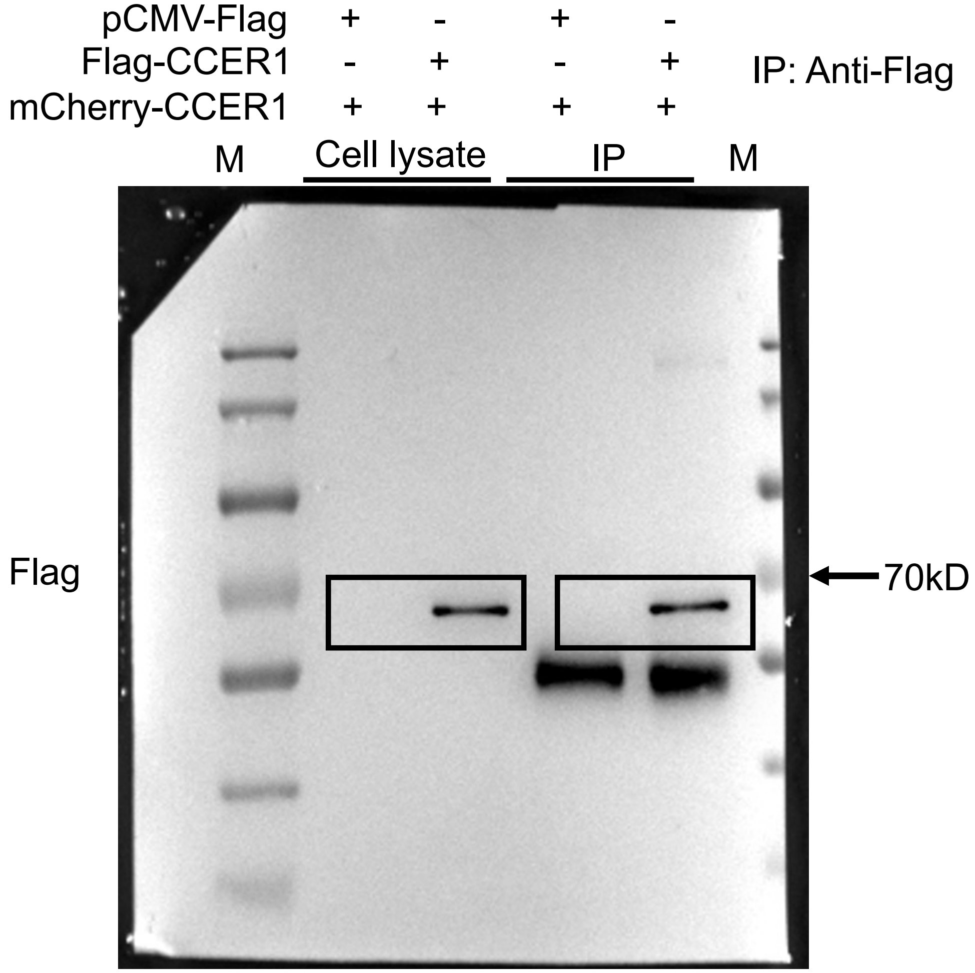

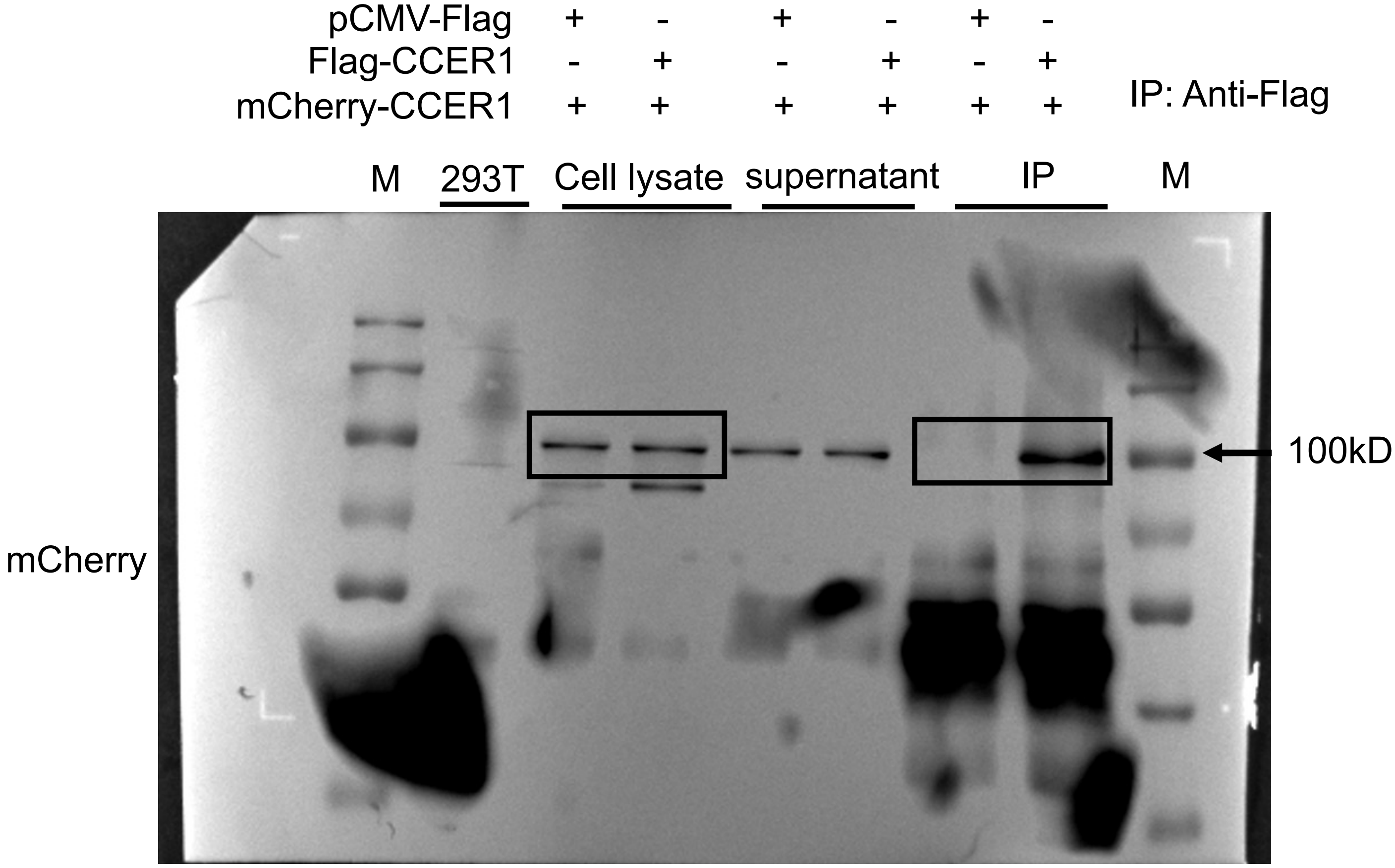

Supplementary Figure 8. Unprocessed images of immunoblotting
PVDF membranes were cut into several small pieces to incubate with different antibodies for immunoblotting. Black boxes indicate images showed in relevant figures.

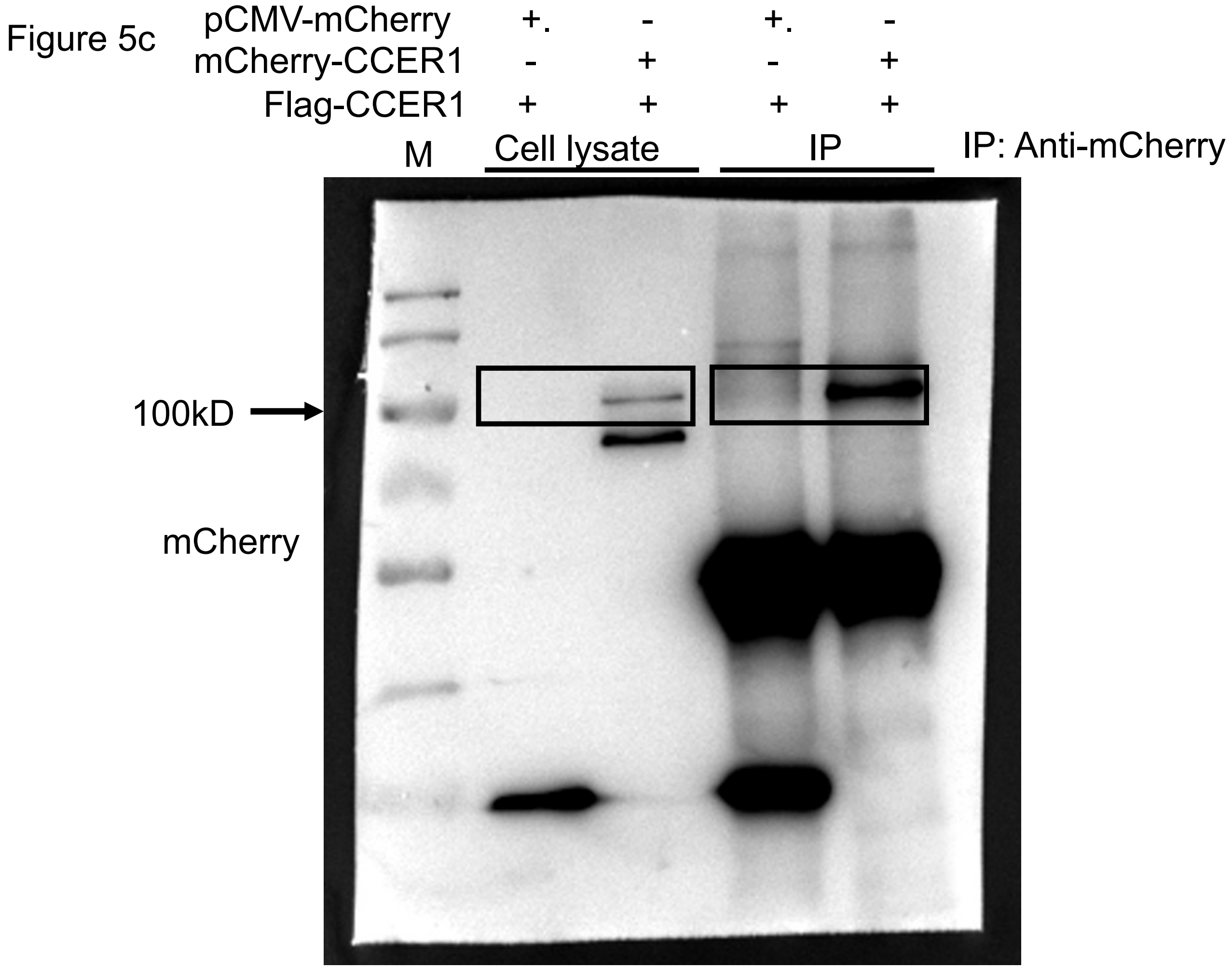

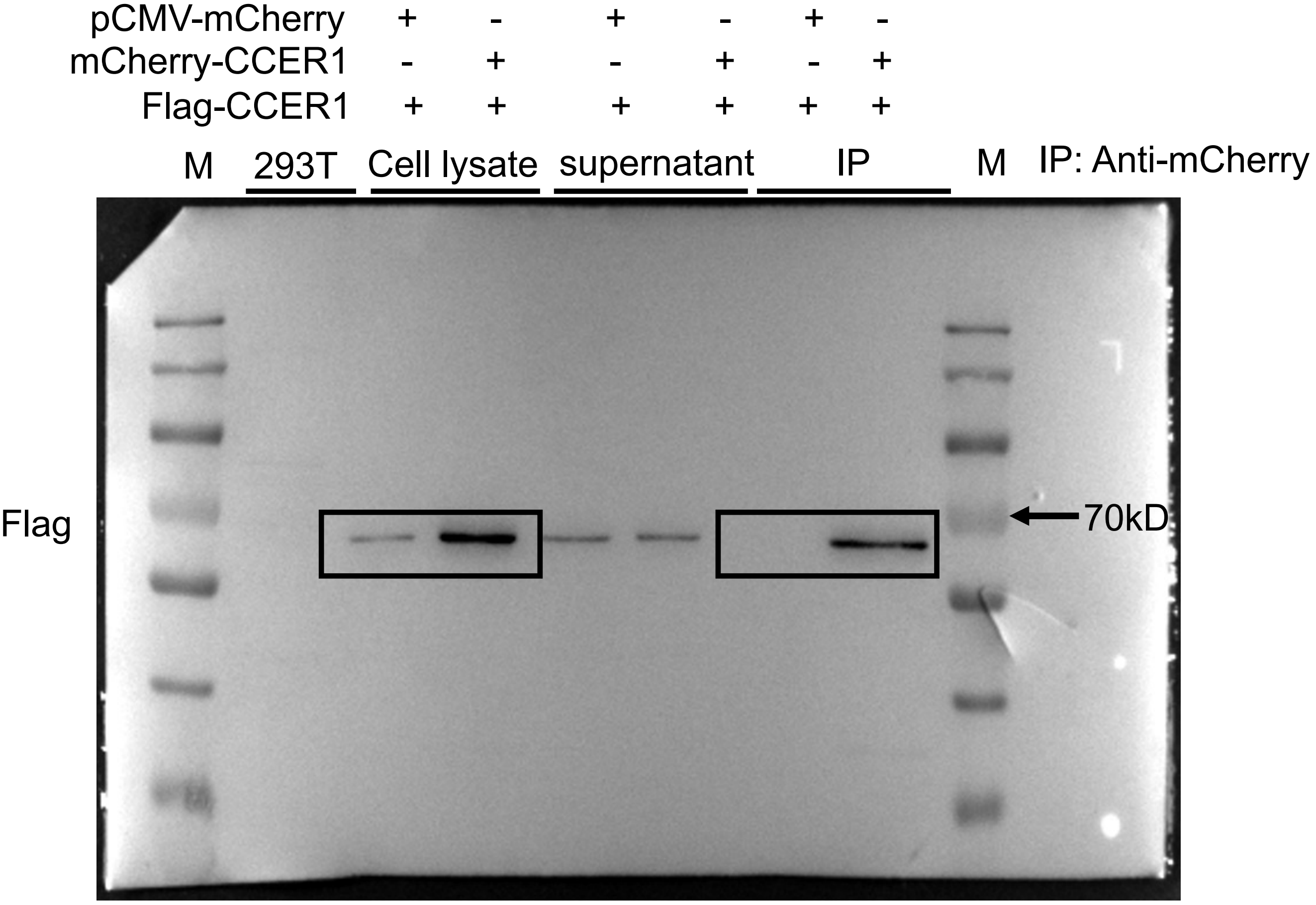

Supplementary Figure 8. Unprocessed images of immunoblotting

PVDF membranes were cut into several small pieces to incubate with different antibodies for immunoblotting. Black boxes indicate images showed in relevant figures.

Figure 5c

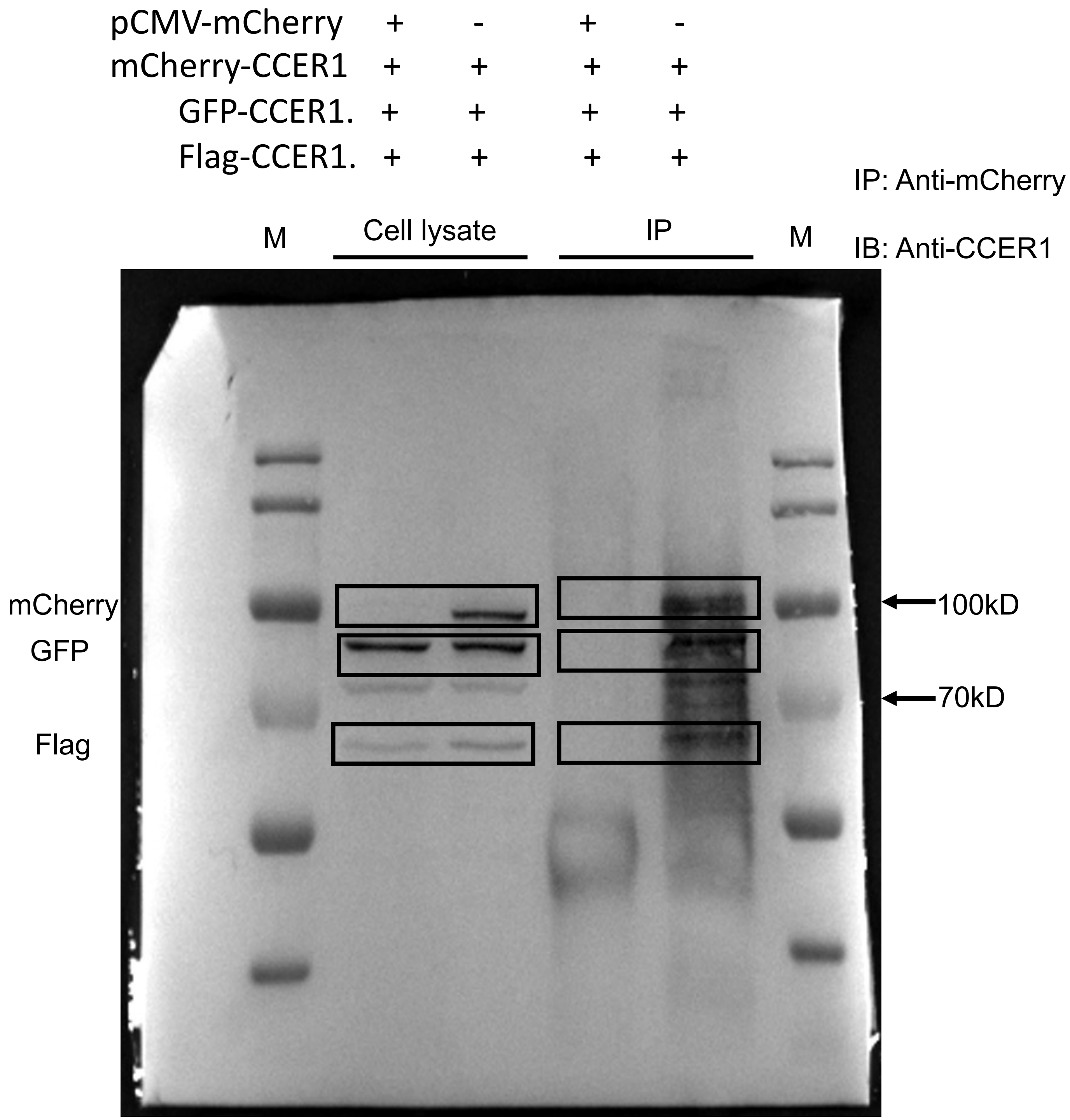

Supplementary Figure 8. Unprocessed images of immunoblotting
PVDF membranes were cut into several small pieces to incubate with different antibodies for immunoblotting. Black boxes indicate images showed in relevant figures.

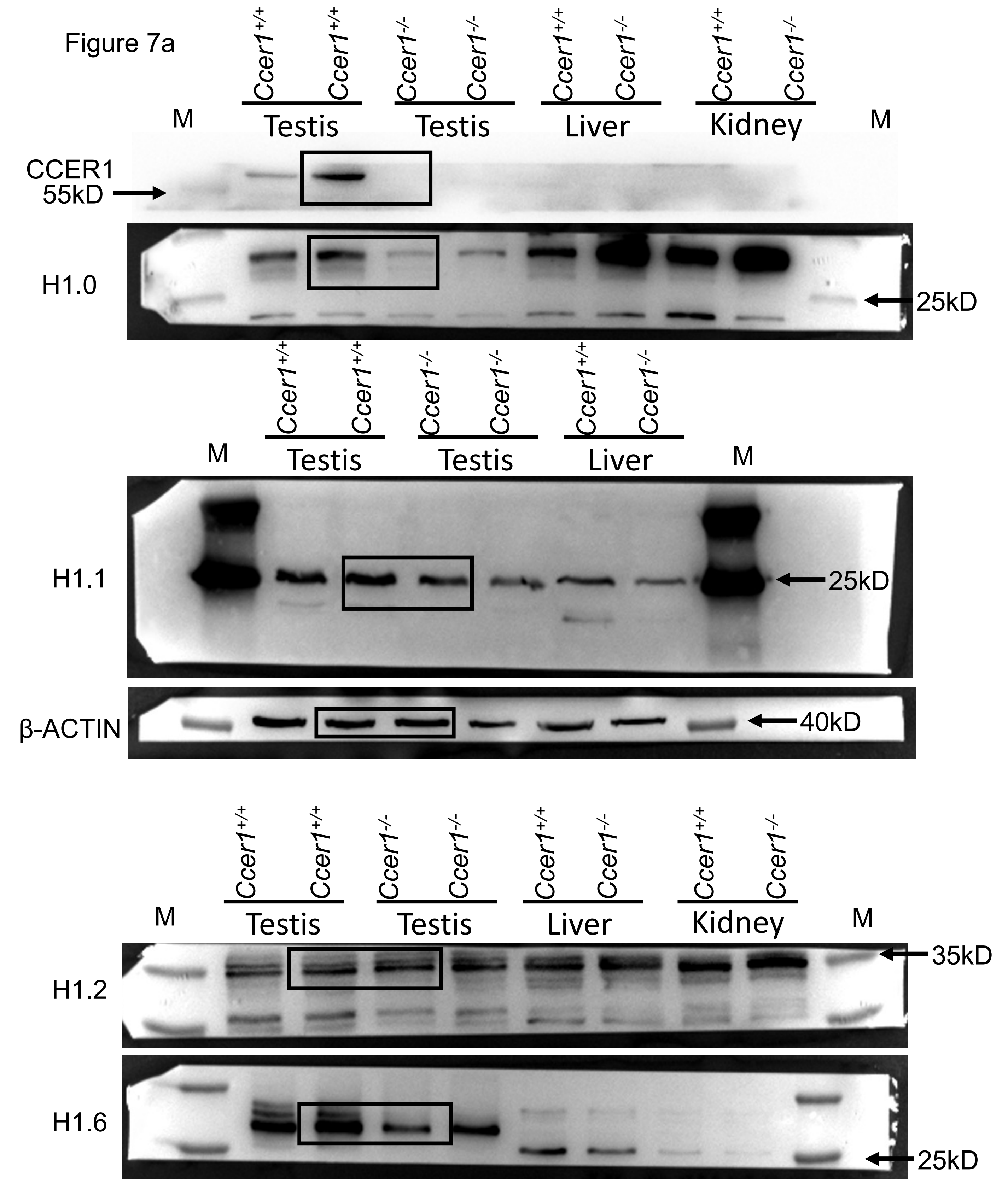

Supplementary Figure 8. Unprocessed images of immunoblotting

PVDF membranes were cut into several small pieces to incubate with different antibodies for immunoblotting. Black boxes indicate images showed in relevant figures.

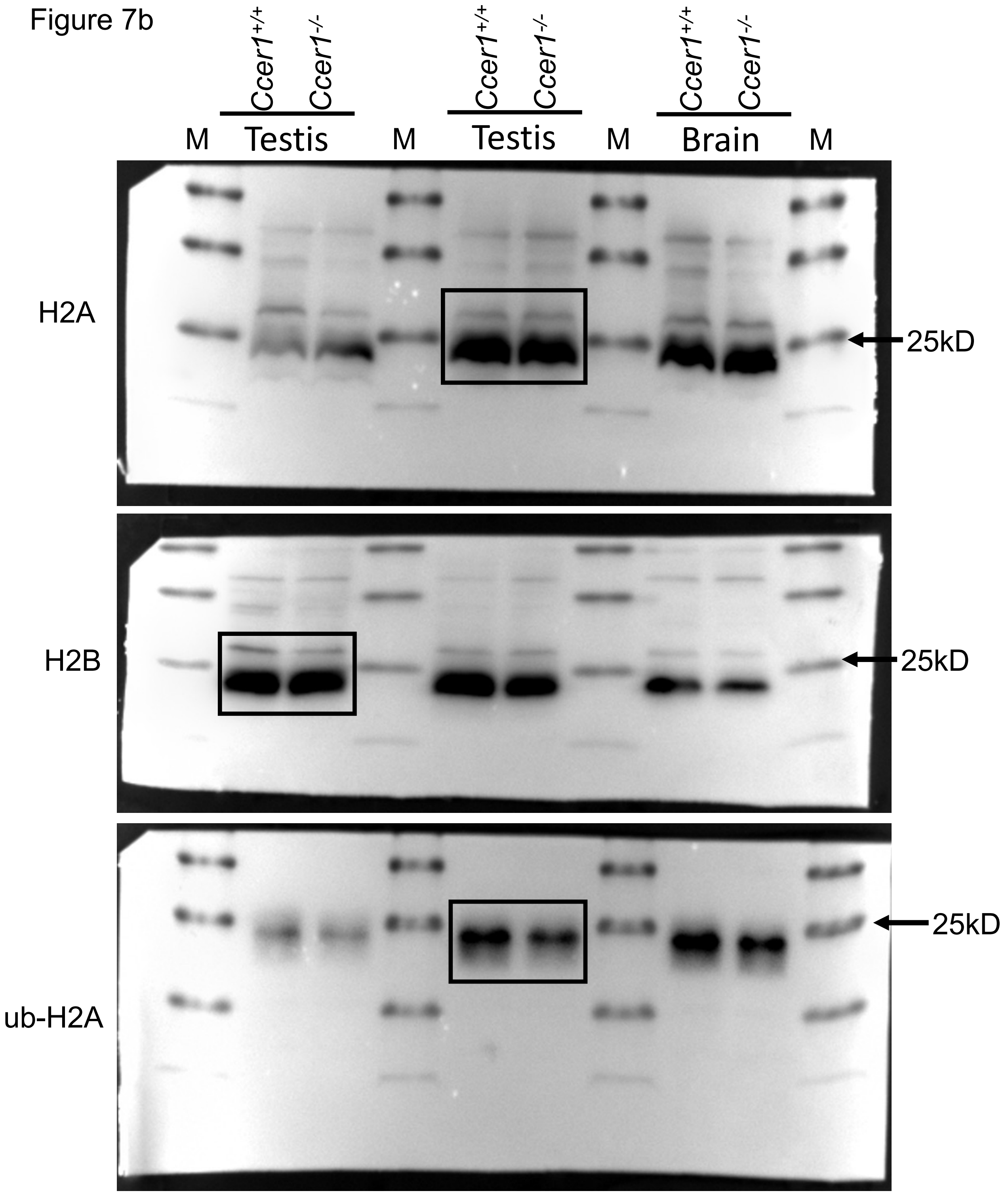

Supplementary Figure 8. Unprocessed images of immunoblotting
PVDF membranes were cut into several small pieces to incubate with different antibodies for immunoblotting. Black boxes indicate images showed in relevant figures.

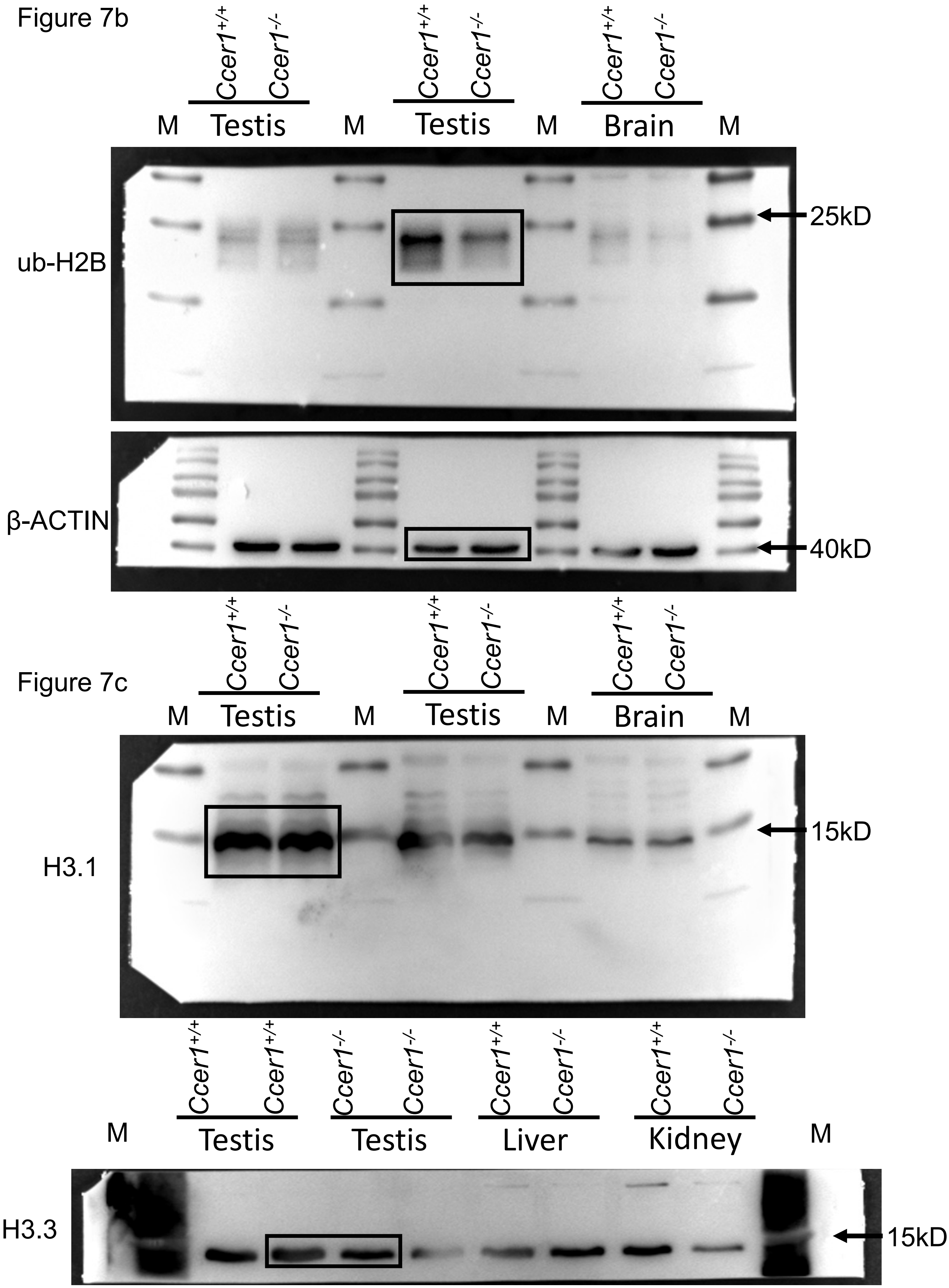

Supplementary Figure 8. Unprocessed images of immunoblotting
PVDF membranes were cut into several small pieces to incubate with different antibodies for immunoblotting. Black boxes indicate images showed in relevant figures.

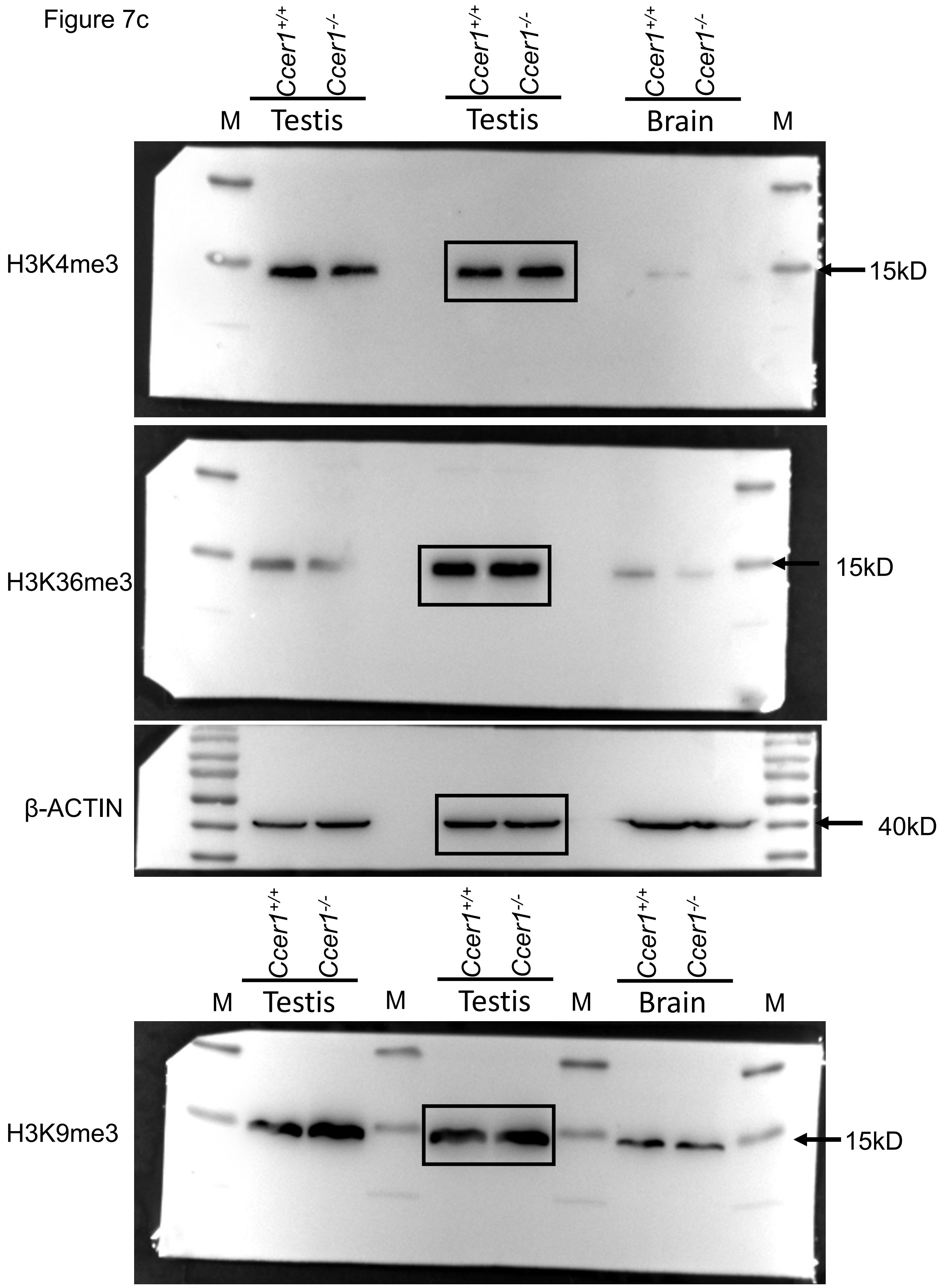

Supplementary Figure 8. Unprocessed images of immunoblotting
PVDF membranes were cut into several small pieces to incubate with different antibodies for immunoblotting. Black boxes indicate images showed in relevant figures.

Figure 7c

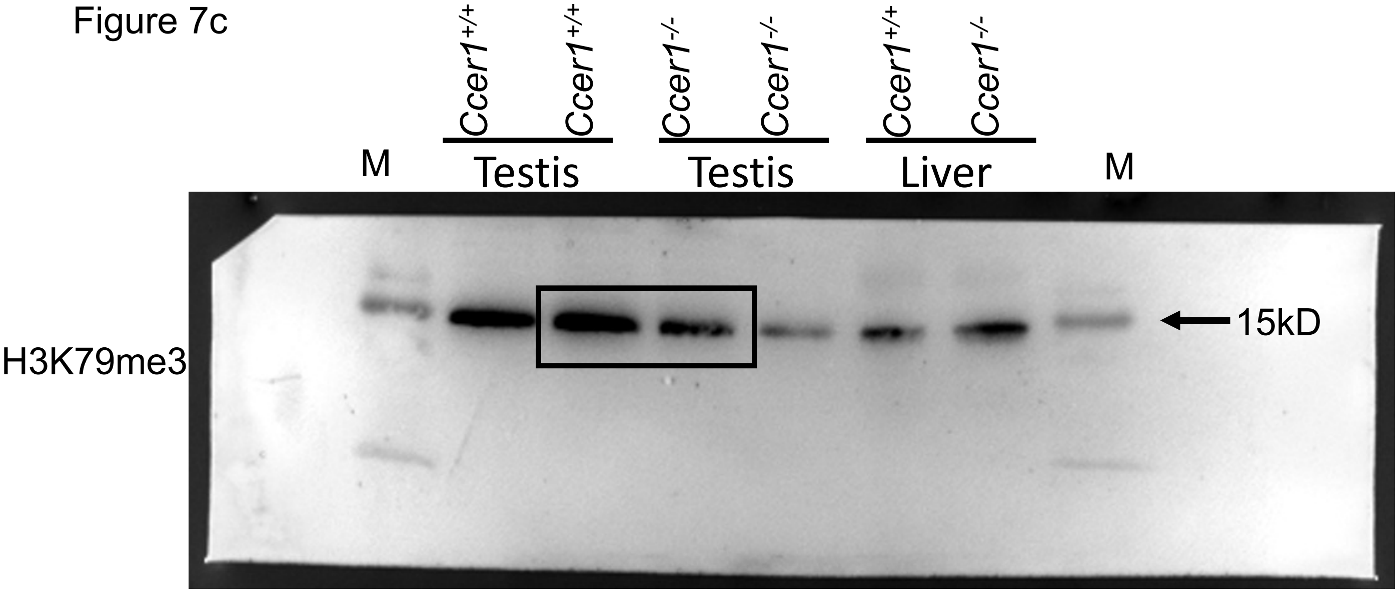

Figure 7d

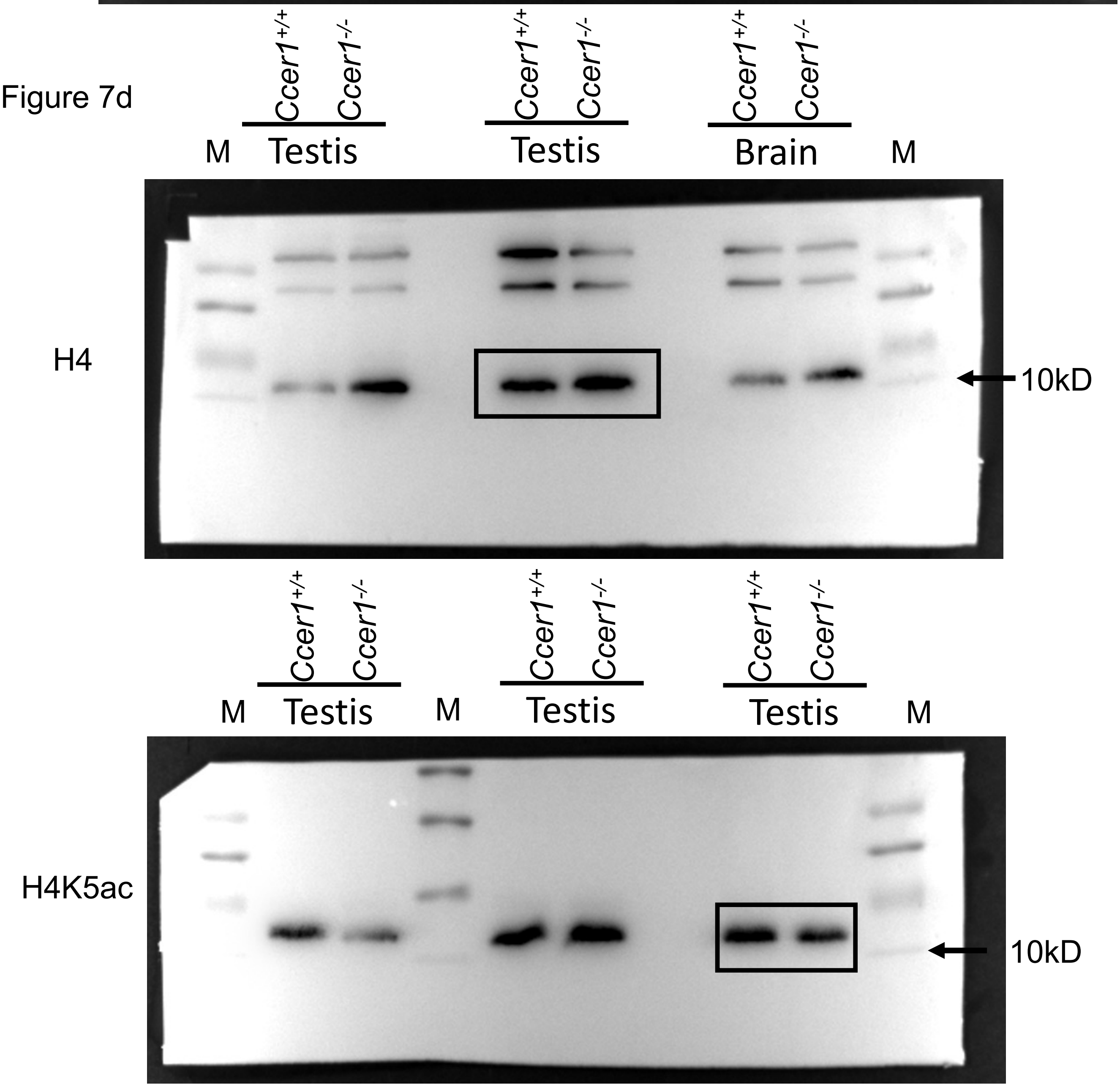

Supplementary Figure 8. Unprocessed images of immunoblotting
PVDF membranes were cut into several small pieces to incubate with different antibodies for immunoblotting. Black boxes indicate images showed in relevant figures.

Figure 7d

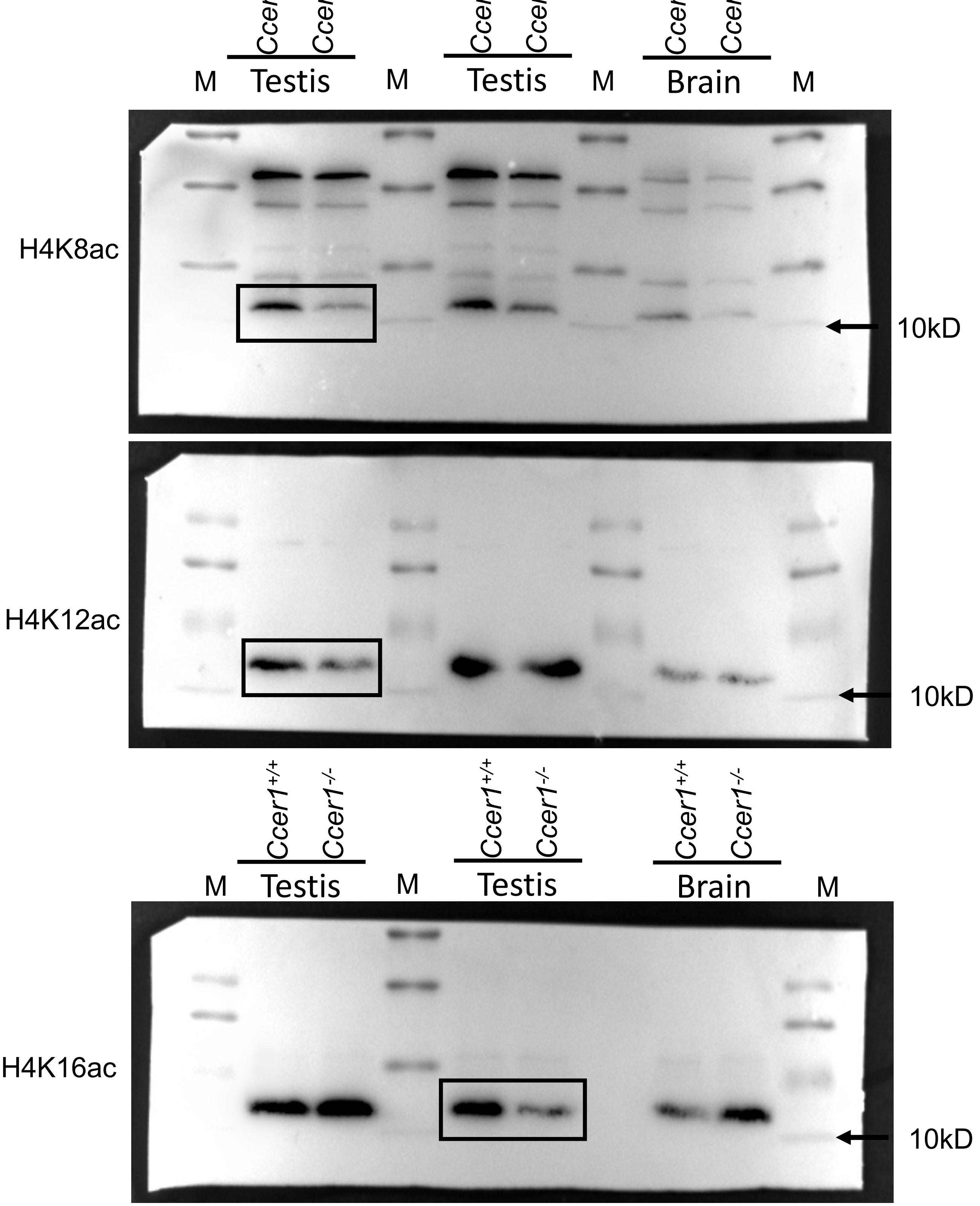

Supplementary Figure 8. Unprocessed images of immunoblotting
PVDF membranes were cut into several small pieces to incubate with different antibodies for immunoblotting. Black boxes indicate images showed in relevant figures.

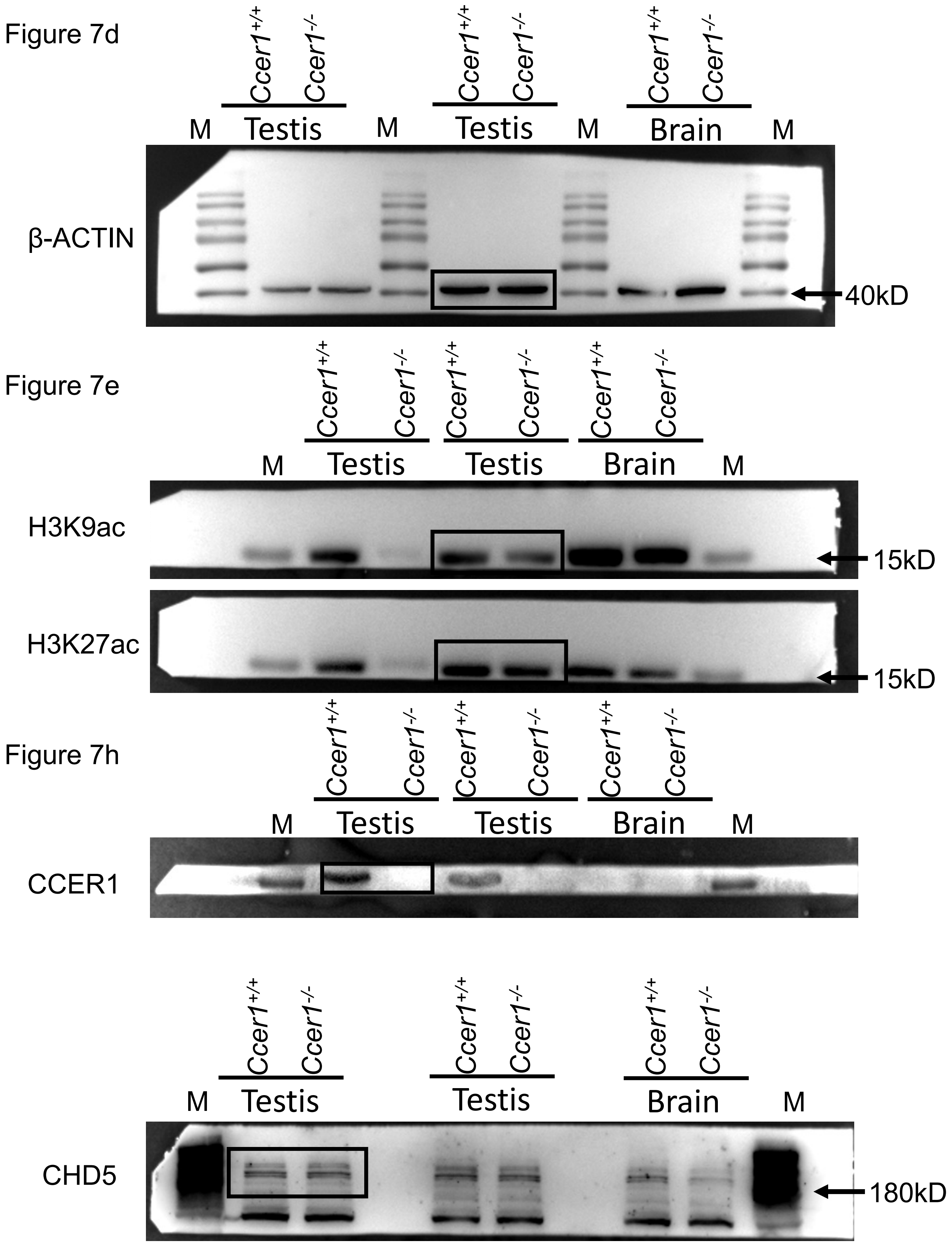

Supplementary Figure 8. Unprocessed images of immunoblotting
PVDF membranes were cut into several small pieces to incubate with different antibodies for immunoblotting. Black boxes indicate images showed in relevant figures.

Figure 7h

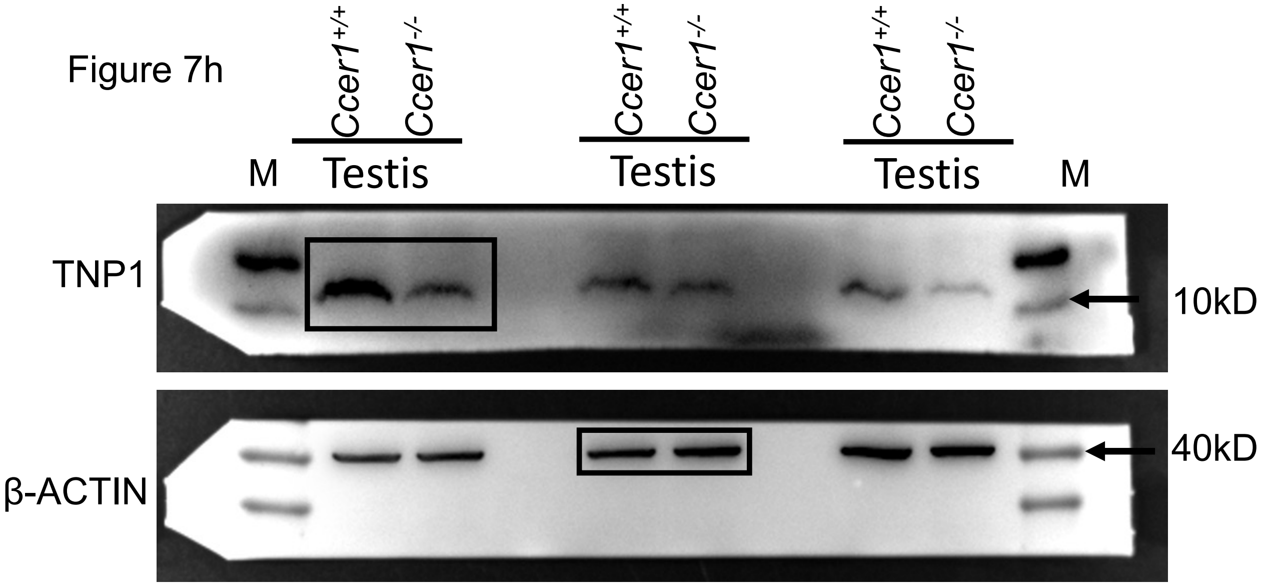

Figure 7i

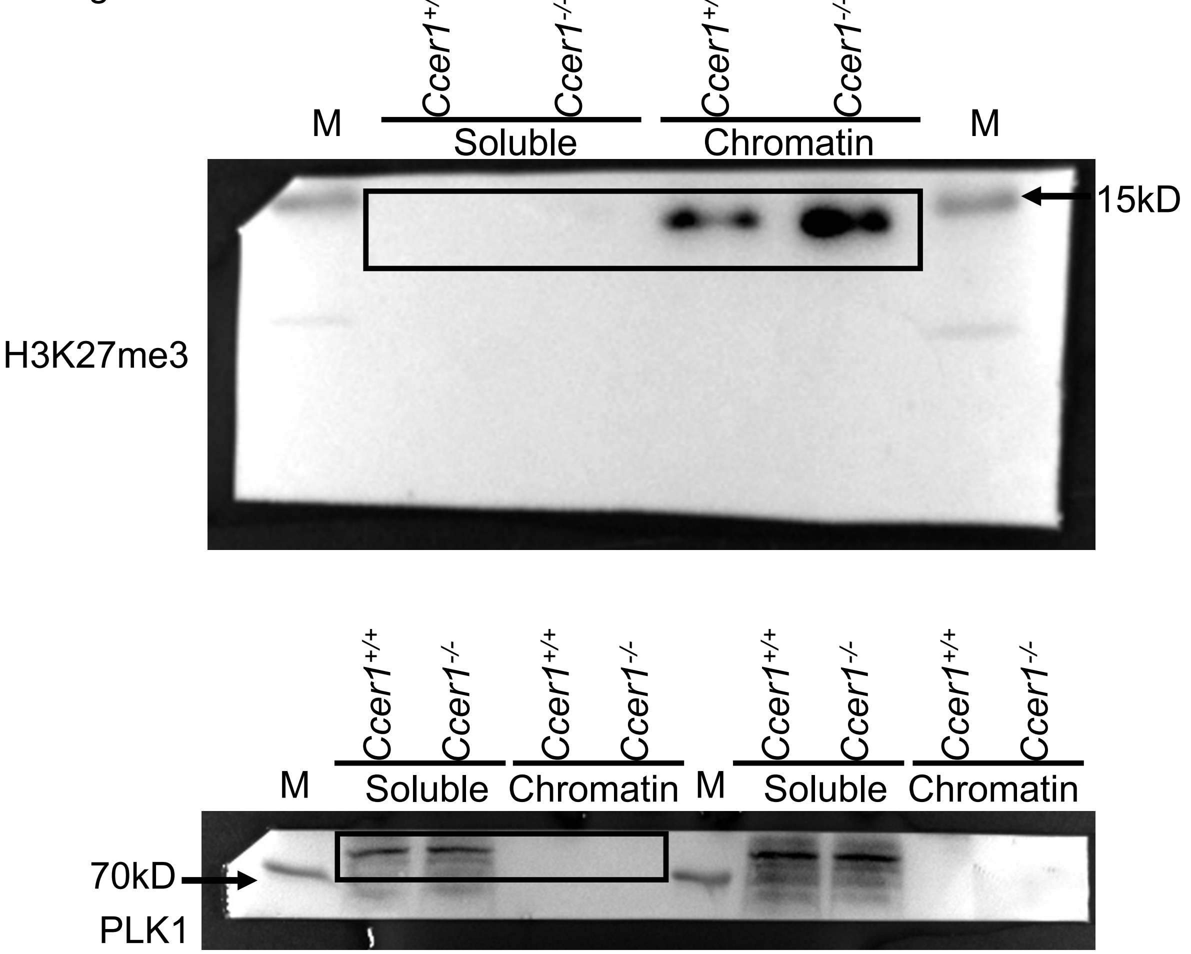

Supplementary Figure 8. Unprocessed images of immunoblotting
PVDF membranes were cut into several small pieces to incubate with different antibodies for immunoblotting. Black boxes indicate images showed in relevant figures.

Figure 7i

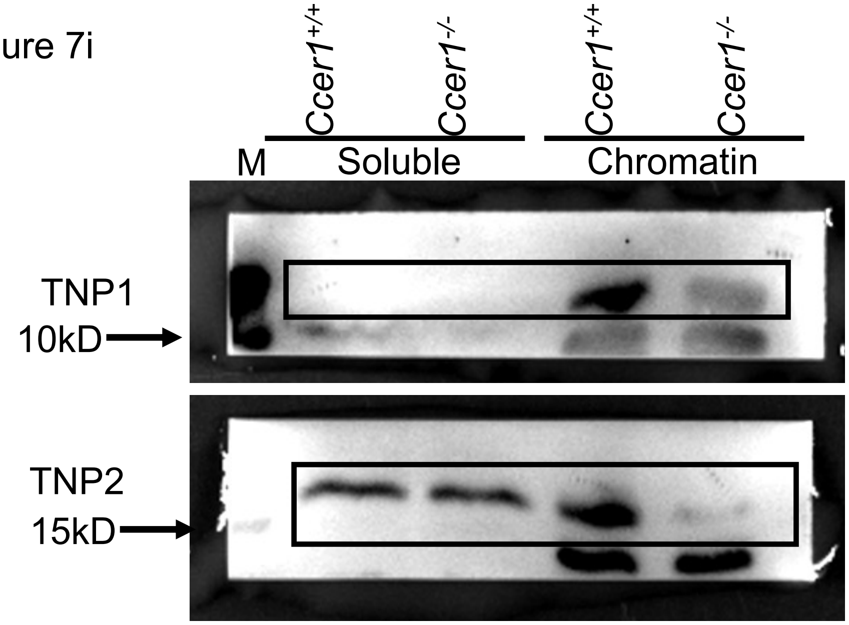

Figure 7j

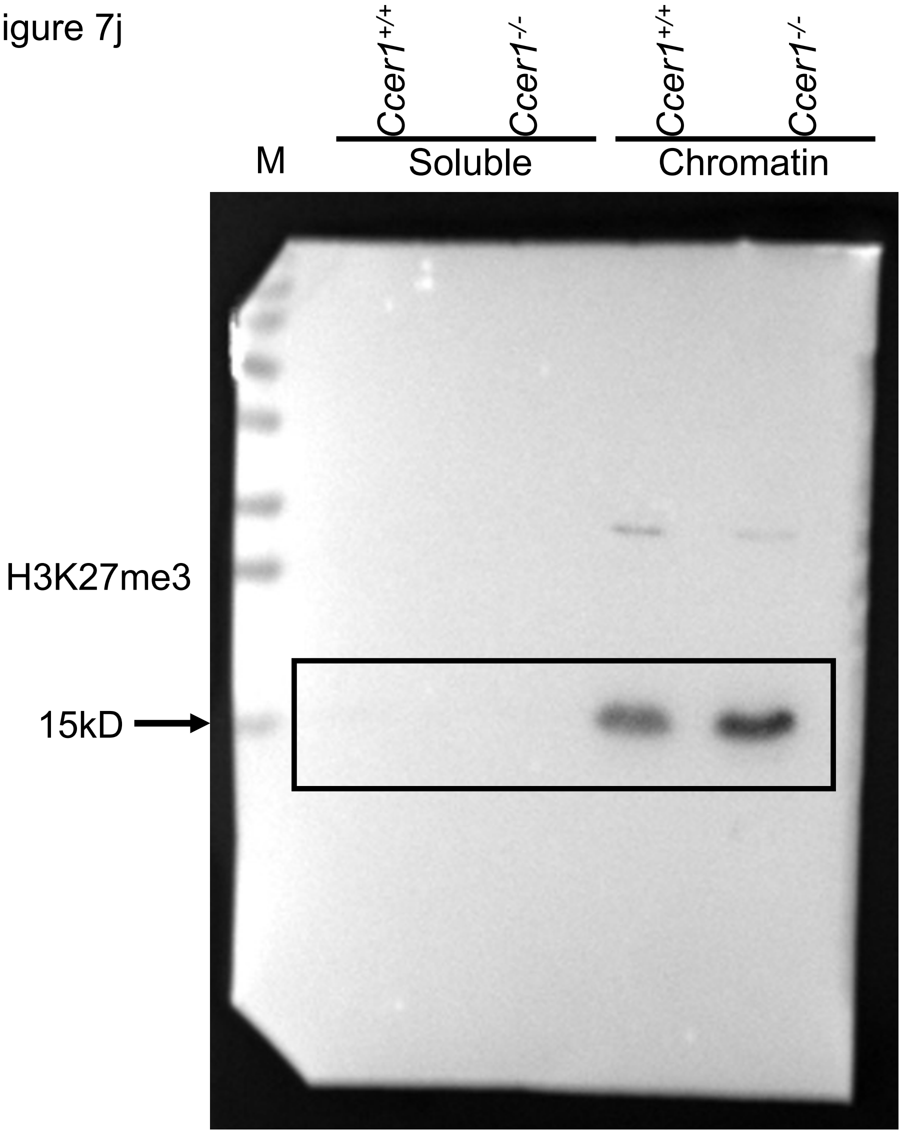

Supplementary Figure 8. Unprocessed images of immunoblotting
PVDF membranes were cut into several small pieces to incubate with different antibodies for immunoblotting. Black boxes indicate images showed in relevant figures.

Figure 7j

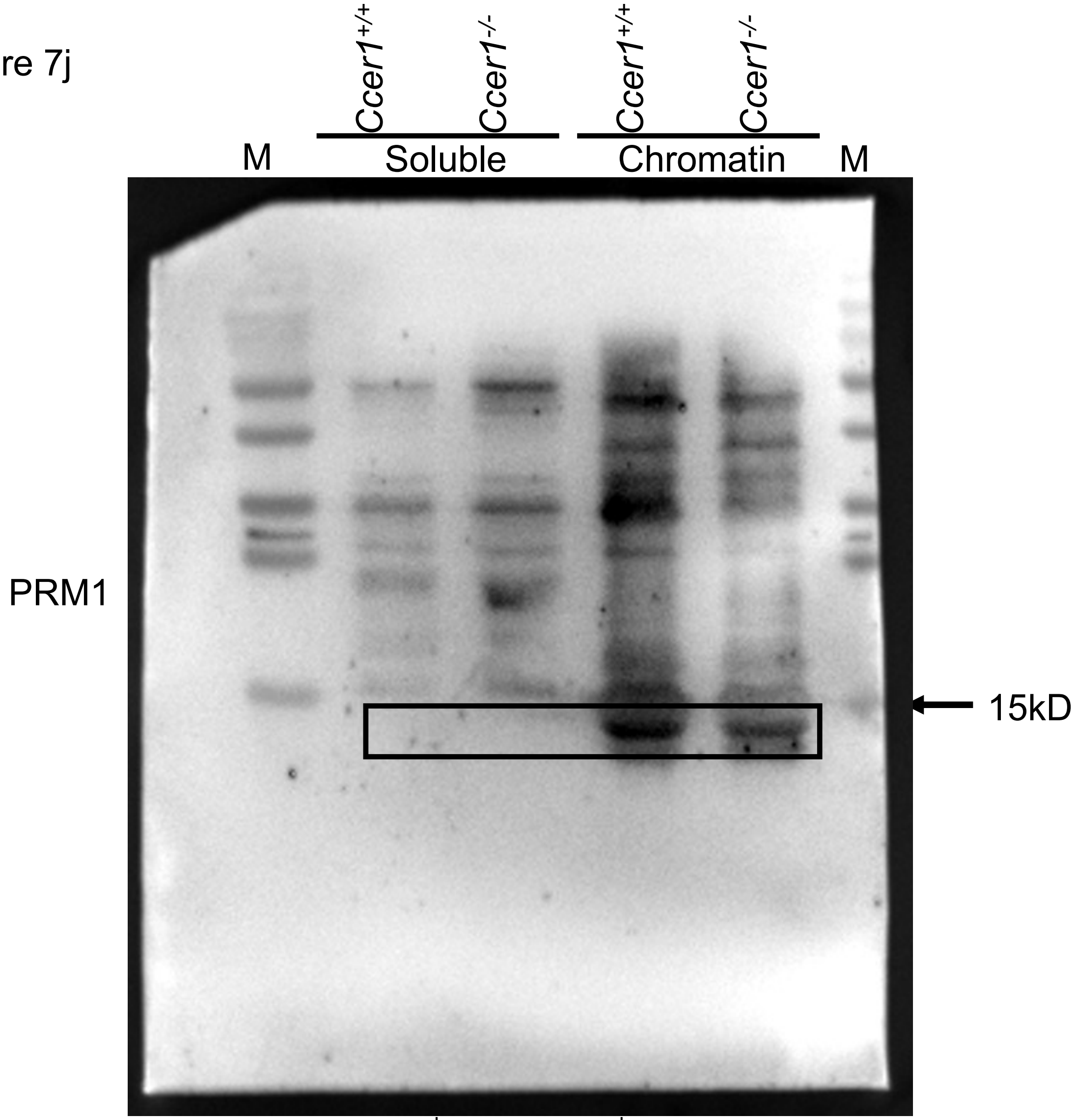

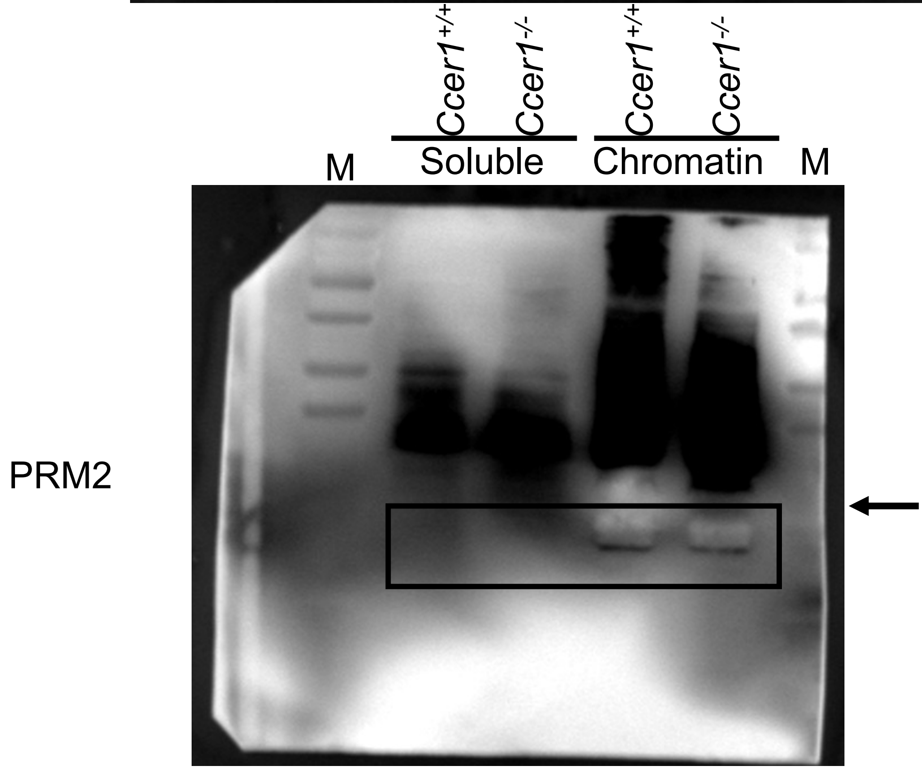

Supplementary Figure 8. Unprocessed images of immunoblotting
PVDF membranes were cut into several small pieces to incubate with different antibodies for immunoblotting. Black boxes indicate images showed in relevant figures.

Supplementary Movie 1. Live-cell imaging shows the dynamic process of EGFP-CCER1 condensate formation.

Supplementary Movie 2. Time course analyses were conducted utilizing confocal laser scanning microscopy and subsequently transformed into video files.

1. Ke Y*, et al.* 3D Chromatin Structures of Mature Gametes and Structural Reprogramming during Mammalian Embryogenesis. *Cell* **170**, 367-381 e320 (2017).

2. Jung YH*, et al.* Maintenance of CTCF- and Transcription Factor-Mediated Interactions from the Gametes to the Early Mouse Embryo. *Mol Cell* **75**, 154-171 e155 (2019).

Supplementary Table1: Key resources used in the research

REAGENT or RESOURCE	SOURCE	IDENTIFIER	Dilution for WB	Dilution for IF/IHC	Dilution for IP
Antibodies					
Rabbit Polyclonal anti-CCER1	Customize in ABclonal Technology (Wuhan,China)		1:2000	1:1000	
Mouse Monoclonal anti-PRM1	Briar Patch Biosciences	Hup1N	1:500		
Mouse Monoclonal anti-PRM1	Abcam	Cat#ab66978	1:500		
Mouse Monoclonal anti-PRM2	Briar Patch Biosciences	Hup2B	1:200	1:100	
Rabbit Polyclonal anti-TNP1	Proteintech	Cat#17178-1-AP	1:1000	1:400	
Mouse Monoclonal anti-TNP2	Santa Cruz	Cat#sc-393843	1:200		
Rabbit Polyclonal anti-TNP2	ABclonal Technology (Wuhan,China)	Cat#A6773	1:500		
Rabbit Polyclonal anti-AKAP3	Proteintech	Cat#13907-1-AP	1:2000		
Rabbit Polyclonal anti-DDX4	Abcam	Cat#ab13840	1:500		
Rabbit Polyclonal anti-CREM	ABclonal Technology (Wuhan,China)	Cat#A5624	1:500		
Rabbit Monoclonal anti-PLK1	ABclonal Technology (Wuhan,China)	Cat#A21082	1:500		
Mouse Monoclonal anti-Flag	Sigma	Cat#F1804	1:1000		1:100
Rabbit Polyclonal anti-GFP	Abcam	Cat#ab290	1:2000		
Rabbit Polyclonal anti-mCherry	Abcam	Cat#ab167453	1:1000		1:100
Mouse Monoclonal anti-β-actin	Sigma	Cat#A1978	1:10000		
Rabbit Polyclonal anti-β-tubulin	Novus Biological	NB600-936	1:5000		
Rabbit Monoclonal anti-Histone H1.0	ABclonal Technology (Wuhan,China)	Cat#A4342	1:500		
Rabbit Polyclonal anti-Histone H1.1	ABclonal Technology (Wuhan,China)	Cat#A20522	1:500		
Rabbit Polyclonal anti-Histone H1.2	Abcam	Cat#ab17677	1:1000		
Rabbit Polyclonal anti-Histone H1.6	ABclonal Technology (Wuhan,China)	Cat#A18597	1:500		
Rabbit Monoclonal anti-Histone H2A	ABclonal Technology (Wuhan,China)	Cat#A3692	1:500		
Rabbit Polyclonal anti-Histone H2B	ABclonal Technology (Wuhan,China)	Cat#A1958	1:500		
Rabbit Polyclonal anti-Histone H3.1	ABclonal Technology (Wuhan,China)	Cat#A2348	1:500		
Rabbit Polyclonal anti-Histone H3.3	ABclonal Technology (Wuhan,China)	Cat#A13824	1:500		
Rabbit Polyclonal anti-Histone H4	ABclonal Technology (Wuhan,China)	Cat#A17024	1:500		
Mouse Monoclonal anti-ub-H2A	Millipore	Cat#05-678	1:1000		
Rabbit Monoclonal anti-ub-H2A	Cell Signaling Technology	Cat#8240	1:1000		
Mouse Polyclonal anti-ub-H2B	MEDIMABS	Cat#MM-0029-P	1:1000		
Rabbit Monoclonal anti-ub-H2B	Cell Signaling Technology	Cat#5546	1:1000		
Mouse Monoclonal anti-H3K4me3	Active Motif	Cat#91263,91264,RRID:AB_2793824	1:1000		
Mouse Monoclonal anti-H3K9me3	Active Motif	Cat#61013,61014,RRID:AB_2687870	1:1000	1:200	
Rabbit Polyclonal anti-H3K9me3	ABclonal Technology (Wuhan,China)	Cat#A2360	1:1000		
Rabbit Polyclonal anti-H3K27me3	Abcam	Cat#ab108245	1:1000		
Rabbit Monolonal anti-H3K36me3	Cell Signaling Technology	Cat#4909	1:1000		
Rabbti Polyclonal anti-H3K79me3	ABclonal Technology (Wuhan,China)	Cat#A2369	1:500		
Rabbit Polyclonal anti-H3K9ac	ABclonal Technology (Wuhan,China)	Cat#A7255	1:500		
Rabbit Monoclonal anti-H3K27ac	Abcam	Cat#ab177178	1:1000		
Rabbit monoclonal anti-H4K5ac	Abcam	Cat#ab51997	1:1000	1:500	
Rabbit Polyclonal anti-H4K8ac	ABclonal Technology (Wuhan,China)	Cat#A7258	1:500	1:500	
Rabbit Polyclonal anti-H4K12ac	ABclonal Technology (Wuhan,China)	Cat#A14227	1:500	1:100	
Rabbit Monoclonal anti-H4K16ac	Abcam	Cat#ab109463	1:1000	1:400	
Rabbit Polyclonal anti-CHD5	ABclonal Technology (Wuhan,China)	Cat#A8578	1:500	1:400	
HRP-conjugated goat anti-rabbit IgG	Zhongshan Jinqiao Biotechnology（China）	Cat#ZB-2301	1:2000		
HRP-conjugated goat anti-mouse IgG	Zhongshan Jinqiao Biotechnology（China）	Cat#ZB-2305	1:2000		
Rhodamine Red™-X -conjugated AffiniPure Donkey Anti-Rabbit IgG（H+L）	Jackson ImmunoResearch	Cat#711-295-152		1:200	
Rhodamine Red™-X -conjugated AffiniPure Donkey Anti-Mouse IgG（H+L）	Jackson ImmunoResearch	Cat#715-025-150		1:200	
Cy™2-conjugated AffiniPure Donkey Anti-Rabbit IgG（H+L）	Jackson ImmunoResearch	Cat#711-225-152		1:200	
Alexa Fluor™ 488 Goat anti-Rabbit IgG（H+L）	Thermo Fisher Scientific	Cat#A11034		1:200	
FITC AffiniPure Donkey Anti-Mouse IgG（H+L）	Jackson ImmunoResearch	Cat#715-095-150		1:200	
Cy™5-conjugated AffiniPure Donkey Anti-Rabbit IgG（H+L）	Jackson ImmunoResearch	Cat#711-175-152		1:200	
DAPI	Sigma	Cat#D9542		1:500	
PNA,Rhodamine	VECTOR LABORATORIES	Cat#RL-1072		1:1000	
PNA,FITC conjugate	Sigma	Cat#L7381		1:1000	
**Recombinant DNA**					
pcDNA3.1(+)-3XFLAG	This paper	N/A			
CpG-free luciferase	PMID: 17965610	N/A			
CpG-free CGI luciferase	This paper	N/A			
Renilla Luc Vector	Invitrogen	Cat# 16154			
mCherry2-C1	gift from Michael Davidson	Addgene plasmid no. 54563			
mCherry2-CCER1	This paper	N/A			
mCherry2-hCCER1	This paper	N/A			
mCherry2-hCCER1-p.Arg53*	This paper	N/A			
mCherry2-hCCER1-p.Cys120fs	This paper	N/A			
mCherry2-hCCER1-p.Trp178	This paper	N/A			
pEGFP-C1	This paper	N/A			
EGFP-CCER1	This paper	N/A			
EGFP-hCCER1	This paper	N/A			
pMaxGFP	Lonza	N/A			
pMaxGFP-CCER1	This paper	N/A			
pMaxGFP-N	This paper	N/A			
pMaxGFP-CC	This paper	N/A			
pMaxGFP-C	This paper	N/A			
pMaxGFP-△CC	This paper	N/A			
Sequence-Based Reagents					
genotyping PCR-F：CCAACTGGGAGCGCTGTGG	This paper				
genotyping PCR-R：GCCACGACCCCTGCGTC	This paper				

qRT-PCR primer

Ccer1-qPCR-F	CCAGGAAACAACCCAAGCA
Ccer1-qPCR-R	AGCAGCAGCAAACGCAGA
Crem-qPCR-F	ATGTCTTGAAAATCGTGTGGCT
Crem-qPCR-R	TGGCAATAAAGGTCTTTGAGGG
Akap3-qPCR-F	ATGGCGGATAGGGTTGACTG
Akap3-qPCR-R	TCGGACAGGATCTGTTGATGT
odf3-qPCR-F	ATGGCAGAGGAGGTATGGATG
odf3-qPCR-R	GGTGGAATCAGGTACTTGGGTC
Tssk6-qPCR-F	CGGGCGACAAACTCCTGAG
Tssk6-qPCR-R	ACCGTCCCTTTATACTTCTTGGA
Spaca9-qPCR-F	AATGAGGTGAAAGAGTCCCTCC
Spaca9-qPCR-R	GGATTTTGTCGTGGGCATTCT
Tnp1-qPCR-F	ACCAGCCGCAAGCTAAAGAC
Tnp1-qPCR-R	TTTCCTACTTTTCAGGACGCTC
Tnp2-qPCR-F	TGAAGCACCCCAAGCCATC
Tnp2-qPCR-R	ATCTTCGCCCTGAGCTACGC
Prm1-qPCR-F	CGAAGATGTCGCAGACGGA
Prm1-qPCR-R	TCACGCAGGAGTTTTGATGG
Prm2-qPCR-F	ATGGTTCGCTACCGAATGAGG
Prm2-qPCR-R	CTCCGCCTTCTGCATGACC

Software and Algorithms

ImageJ	NIH	https://imagej.en.softonic.com/
PONDR		http://www.pondr.com/
GraphPad Prism	GraphPad Software	https://www.graphpad.com/
Photoshop CS5	Adobe	https://www.adobe.com/
Adobe illustrator	Adobe	https://www.adobe.com/
