# Supplementary material for: Phase-separated CCER1 coordinates the histone-to-protamine transition and male fertility
Source: Nat Commun. 2023 Dec 11;14:8209. doi: 10.1038/s41467-023-43480-z (PMC10713660; doi:10.1038/s41467-023-43480-z)
Supplement: Supplementary file 3 — Supplementary Movie [file 41467_2023_43480_MOESM3_ESM.pdf]

Description of Additional Supplementary Files

File Name: Supplementary Movie 1
Description: Live-cell imaging shows the dynamic process of EGFP-CCER1 condensate formation.

File Name: Supplementary Movie 2
Description: Live-cell imaging shows the dynamic process of EGFP-CCER1 condensate disruption after 10% 1,6-hexanediol treatment.
